# Comparative Analysis of Genome of *Ehrlichia* sp. HF, a Model Bacterium to Study Fatal Human Ehrlichiosis

**DOI:** 10.1186/s12864-020-07309-z

**Published:** 2021-01-06

**Authors:** Mingqun Lin, Qingming Xiong, Matthew Chung, Sean C. Daugherty, Sushma Nagaraj, Naomi Sengamalay, Sandra Ott, Al Godinez, Luke J. Tallon, Lisa Sadzewicz, Claire Fraser, Julie C. Dunning Hotopp, Yasuko Rikihisa

**Affiliations:** 1 Department of Veterinary Biosciences, The Ohio State University, 1925 Coffey Road, Columbus, OH 43210 USA; 2 Institute for Genome Sciences, University of Maryland School of Medicine, 801 W. Baltimore St, Baltimore, MD 21201 USA; 3 Department of Medicine, University of Maryland School of Medicine, 801 W. Baltimore St, Baltimore, MD 21201 USA; 4 Department of Microbiology and Immunology, University of Maryland School of Medicine, 801 W. Baltimore St, Baltimore, MD 21201 USA; 5 Greenebaum Cancer Center, University of Maryland School of Medicine, 801 W. Baltimore St, Baltimore, MD 21201 USA

**Keywords:** *Ehrlichia* sp. HF, Monocytic Ehrlichiosis, Mouse model, Comparative genomic analysis, Core genome alignment, Virulence factors

## Abstract

**Background:**

The genus *Ehrlichia* consists of tick-borne obligatory intracellular bacteria that can cause deadly diseases of medical and agricultural importance. *Ehrlichia* sp. HF, isolated from *Ixodes ovatus* ticks in Japan [also referred to as *I. ovatus Ehrlichia* (IOE) agent], causes acute fatal infection in laboratory mice that resembles acute fatal human monocytic ehrlichiosis caused by *Ehrlichia chaffeensis*. As there is no small laboratory animal model to study fatal human ehrlichiosis, *Ehrlichia* sp. HF provides a needed disease model. However, the inability to culture *Ehrlichia* sp. HF and the lack of genomic information have been a barrier to advance this animal model. In addition, *Ehrlichia* sp. HF has several designations in the literature as it lacks a taxonomically recognized name.

**Results:**

We stably cultured *Ehrlichia* sp. HF in canine histiocytic leukemia DH82 cells from the HF strain-infected mice, and determined its complete genome sequence. *Ehrlichia* sp. HF has a single double-stranded circular chromosome of 1,148,904 bp, which encodes 866 proteins with a similar metabolic potential as *E. chaffeensis*. *Ehrlichia* sp. HF encodes homologs of all virulence factors identified in *E. chaffeensis*, including 23 paralogs of P28/OMP-1 family outer membrane proteins, type IV secretion system apparatus and effector proteins, two-component systems, ankyrin-repeat proteins, and tandem repeat proteins. *Ehrlichia* sp. HF is a novel species in the genus *Ehrlichia*, as demonstrated through whole genome comparisons with six representative *Ehrlichia* species, subspecies, and strains, using average nucleotide identity, digital DNA-DNA hybridization, and core genome alignment sequence identity.

**Conclusions:**

The genome of *Ehrlichia* sp. HF encodes all known virulence factors found in *E. chaffeensis*, substantiating it as a model *Ehrlichia* species to study fatal human ehrlichiosis. Comparisons between *Ehrlichia* sp. HF and *E. chaffeensis* will enable identification of *in vivo* virulence factors that are related to host specificity, disease severity, and host inflammatory responses. We propose to name *Ehrlichia* sp. HF as *Ehrlichia japonica* sp. nov. (type strain HF), to denote the geographic region where this bacterium was initially isolated.

**Supplementary Information:**

The online version contains supplementary material available at 10.1186/s12864-020-07309-z.

## Background

The incidence of tick-borne diseases has risen dramatically in the past two decades, and continues to rise [1–3]. The 2011 Institute of Medicine report “*Critical Needs and Gaps in...Lyme and Other Tick-Borne Diseases*” revealed the urgent need for research into tick-borne diseases [4]. *Ehrlichia* species are tick-borne obligate intracellular bacteria, which are maintained via the natural transmission and infection cycle between particular species of ticks and mammals (Table 1). The genus *Ehrlichia* belongs to the family Anaplasmataceae in the order Rickettsiales. According to International Code of Nomenclature of Prokaryotes and International Journal of Systematic and Evolutionary Microbiology [46], and following the reorganization of genera in the family Anaplasmataceae based on molecular phylogenetic analysis [47], the genus *Ehrlichia* currently consists of six taxonomically classified species with validly published names, including *E. chaffeensis*, *E. ewingii*, *E. canis*, *E. muris*, *E. ruminantium*, and a recently culture-isolated *E. minasensis* that is closely related to *E. canis* (Table 1) [19, 37]. Accidental transmission and infection of domestic animals and humans can cause potentially severe to fatal diseases, and four species (*E. chaffeensis*, *E. ewingii*, *E. canis*, and *E. muris*) are known to infect humans and cause emerging tick-borne zoonoses [11, 19–21, 34, 48, 49]. In the US, the most common human ehrlichiosis is human monocytic ehrlichiosis (HME) caused by *E. chaffeensis,* which was discovered in 1986 [12]*,* followed by human Ewingii ehrlichiosis discovered in 1998 [34]. The most recently discovered human ehrlichiosis is caused by *E. muris* subsp. *eauclairensis* [originally referred to as *E. muris*-like agent (EMLA)] [19, 20]. Human infection with *E. canis* has been reported in South and Central America [21, 22, 49]. Regardless of the *Ehrlichia* species, clinical signs of human ehrlichiosis include fever, headache, myalgia, thrombocytopenia, leukopenia, and elevated serum liver enzyme levels [20, 21, 34, 48–50].

**Table 1:** Biological characteristics of representative *Ehrlichia* species

Species^1^ (Type strain)	Diseases	Mammalian Host	Tick Vector/Host	Geographic Distribution	References
*Ehrlichia.* sp. HF (HF565)	Acute fatal infection of mice (experimental)	Unknown	*Ixodes ovatus* , *I. ricinus*, and *I. apronophorus* ticks	Japan, France, Serbia, Romania	[5–10]
*E. chaffeensis* (Arkansas)^2^	Human monocytic ehrlichiosis (HME)	Deer, Human, Dog, Coyote, Fox^3^	*Amblyomma americanum* (Lone star tick)	USA, Africa, South America, Europe, Japan	[11–16]
*E. muris* subsp. *muris* (AS145)	Murine monocytic ehrlichiosis (chronic systemic infection of mice)	Mouse, Vole	Ticks (*Haemaphysalis flava* or *Ixodes persulcatus*)	Japan, Russia^4^	[17, 18]
*E. muris* subsp. *eauclairensis* (Wisconsin)	Human or murine monocytic ehrlichiosis (fatal infection of mice)	Human, Mouse	*Ixodes scapularis* (black-legged tick)	Wisconsin and Minnesota, USA	[19, 20]
*E. canis* (Oklahoma)	Canine tropical pancytopenia, Venezuelan Human Ehrlichiosis ^5^	Dog, Human	*Rhipicephalus sanguineus* (brown dog tick)	Global	[21–25]
*E. ruminantium* (Welgevonden)	Heartwater	Ruminants (Cattle, Sheep, Goats, Antelope)	Various *Amblyomma* species of ticks	Africa, Caribbean^6^	[26–33]
*E. ewingii* (Stillwater)	Canine granulocytic ehrlichiosis, Human ewingii ehrlichiosis	Deer, Dog, Human	*Amblyomma americanum*	USA, Japan	[34–36]
*E. minasensis* (UFMG-EV)	Ehrlichiosis	Cattle, Deer, Dog ^7^	*Rhipicephalus microplus* tick	Brazil, Global	[37–45]

^1^Based on International Code of Nomenclature of Prokaryotes, and published in International Journal of Systematic and Evolutionary Microbiology, which lists officially approved list of bacterial classification and nomenclature, the genus *Ehrlichia* currently consists of six validly published species with correct names (https://lpsn.dsmz.de/genus/ehrlichia)

^2^*Ehrlichia* sp. HF, or *Ixodes ovatus Ehrlichia* (IOE) agent, is a field tick isolate of *Ehrlichia* species in Fukushima Prefecture, Japan from 1993 to 1994. *Ehrlichia* sp. HF DNA was also detected in *I. ricinus* tick from Brittany, France and Serbia, and *I. apronophorus* tick in Romania

^3^*E. chaffeensis* DNA was detected in 71% of free-ranging coyotes in Oklahoma and experimentally infected red foxes

^4^*E muris* DNA was found in *I. persulcatus* ticks and small mammals in Russia

^5^Human Infection with *E. canis* with clinical signs was reported in Venezuela, and *E. canis* was culture isolated from a VHE patient. In addition, *E. canis* DNA was detected in human blood bank donors in Costa Rica

^6^Heartwater in Caribbean islands of Guadeloupe was caused *E. ruminantium* Gardel, which is transmitted by *Amblyomma variegatum* (Tropical bont tick) and exceptionally virulent in Dutch goats. More heartwater cases in wild and domestic ruminants have been reported in five Caribbean islands, posing an increasing threat to domestic and wild ruminants in the continental US

^7^*E. minasensis* strain UFMG-EVT was isolated from the haemolymph of engorged *Rhipicephalus microplus* female ticks in Brazil, whereas strain Cuiaba was isolated from the whole blood of a naturally infected cattle. *E. minasensis* DNAs have also been reported in ticks, cervids, and dogs from France, Pakistan, Ethiopia, and Israel

HME is a significant, emerging tick-borne disease with serious health impacts with the highest incidence in people over 60 years of age and immunocompromised individuals [48]. Life-threatening complications such as renal failure, adult respiratory distress syndrome, meningoencephalitis, multi-system organ failure, and toxic shock occur in a substantial portion of the patients who are hospitalized and resulting in a case fatality rate of 3% [48]. However, there is no vaccine available for HME [51], and the only drug of choice is doxycycline, which is only effective with early diagnosis and treatment, and is not suitable for all patient groups [48]. In addition, pathogenesis and immunologic studies on human ehrlichiosis have been hampered due to the lack of an appropriate small animal disease model, as *E. chaffeensis* only transiently infects immunocompetent laboratory mice [52, 53]. *E. chaffeensis* naturally infects dogs and deer with mild to no clinical signs [53–55]. However, use of these animals is difficult and cost-prohibitive, while not being suitable for pathogenesis studies.

In an attempt to determine the pathogens harbored by *Ixodes ovatus* ticks prevalent in Japan, Fujita and Watanabe inoculated tick homogenates into the intraperitoneal cavity of laboratory mice, followed by serial passage through naïve mice using homogenized spleens from infected mice [5]. From 1983 to 1994, twelve “HF strains” were isolated from *I. ovatus* ticks in this manner, with the strain named after the scientist Hiromi Fujita who first discovered and isolated this bacterium [5]. Electron micrographs of HF326 showed the typical ultrastructure of *Ehrlichia* in the mouse liver [5]. A few years later, analysis of the 16S rRNA gene of the HF strains showed that four isolates (HF565, HF568-1, HF568-2, and HF639-2) from Fukushima, and two isolates (HF642 and HF652) from Aomori, northern Japan, were identical and closely related to *Ehrlichia* spp. [6]. The phylogenetic comparison of 16S rRNA and GroEL protein sequences of HF565 with those of members of the family *Anaplasmataceae,* and electron micrographs of HF565 verified that the HF strain belongs to the genus *Ehrlichia* [6]. Recent studies indicated that DNA sequences of *Ehrlichia* sp. HF have been detected not only in *I. ovatus* ticks throughout Japan, but also in *Ixodes ricinus* ticks in France [7] and Serbia [8], and *Ixodes apronophorus* ticks in Romania [9].

Unlike *E. muris*, HF565 does not induce splenomegaly but is highly virulent in mice, as intraperitoneal inoculation kills immunocompetent laboratory mice in 6-10 days [5, 6, 10, 56]. HF565 (the HF strain described here) was requested by and distributed to several US laboratories, where the strain was dubbed as *I. ovatus Ehrlichia* (IOE) agent. Using the HF strain-infected mouse spleen homogenate as the source of HF bacterium, pathogenesis studies in inoculated mice revealed that these bacteria induce a toxic shock-like cytokine storm, involving cytotoxic T-cells, NKT cells, and neutrophils similar to those reported in fatal HME [57–68]. Therefore, *Ehrlichia* sp. HF has been increasingly serving as a needed immunocompetent mouse model for studying fatal ehrlichiosis.

The major barriers for advancing research on *Ehrlichia* sp. HF, however, have been the inability to stably culture it in a mammalian macrophage cell line and lack of genome sequence and analysis data. Previously, it was cultured in monkey endothelial RF/6A cells and *Ixodes scapularis* tick embryo ISE6 cells [69]. To facilitate studies using *Ehrlichia* sp. HF, we stably cultured the HF strain in a canine histiocytic leukemia cell line DH82, and obtained the complete whole genome sequence (GenBank accession NZ_CP007474). Despite many studies being conducted with *Ehrlichia* sp. HF, this bacterium has not been classified into any species, causing confusion in the literature with several different names (IOE agent, *Ehrlichia* sp. HF, the HF strain). Comparative core genome alignment and phylogenetic analysis reveal that *Ehrlichia* sp. HF is a new species that is most closely related to *E. muris* and *E. chaffeensis,* justifying the formal nomenclature of this species. The genome sequencing and analysis, including comparative virulence factor analysis of *Ehrlichia* sp. HF, provides important insights, resources, and validation for advancing the research on emerging human ehrlichioses.

## Results and Discussion

### Culture Isolation of *Ehrlichia* sp. HF and purification of *Ehrlichia* genomic DNA

To obtain sufficient amounts of bacterial DNA free from host cell DNA, we stably cultured *Ehrlichia* sp. HF in DH82 cells. Spleen and blood samples were collected from *Ehrlichia* sp. HF-infected mice euthanized at an acute stage of illness (8 d post inoculation) (Fig. S1A). Diff-Quik staining showed that the bacteria were present in blood monocytes (Fig. S1B). After 2 - 3 weeks co-culturing with infected spleen homogenates, large vacuoles (inclusions) containing numerous bacteria (known as morulae) were observed in the cytoplasm of DH82 (Fig. S1C) and RF/6A cells (Fig. S1D). *Ehrlichia* sp. HF could also be successfully passaged from DH82 cells to ISE6 cells (Fig. S1E). Morulae of *Ehrlichia* sp. HF in cell cultures were like those seen in the tissue sections of the thymus and the lungs of infected mice [6], and in the endothelial cells of most organs of infected mice [10]. *Ehrlichia* sp. HF cultured in DH82 cells infects and kills mice at 7 – 10 days post intraperitoneal inoculation, similar to those inoculated with the infected mouse spleen homogenate, demonstrating that *Ehrlichia* sp. HF culture isolate maintains mouse virulence [56]. The mouse LD_50_ of *Ehrlichia* sp. HF cultured in DH82 cells is approximately 100 bacteria [56].

### General features of the ***Ehrlichia*sp. HF** genome

**Fig. 1:**
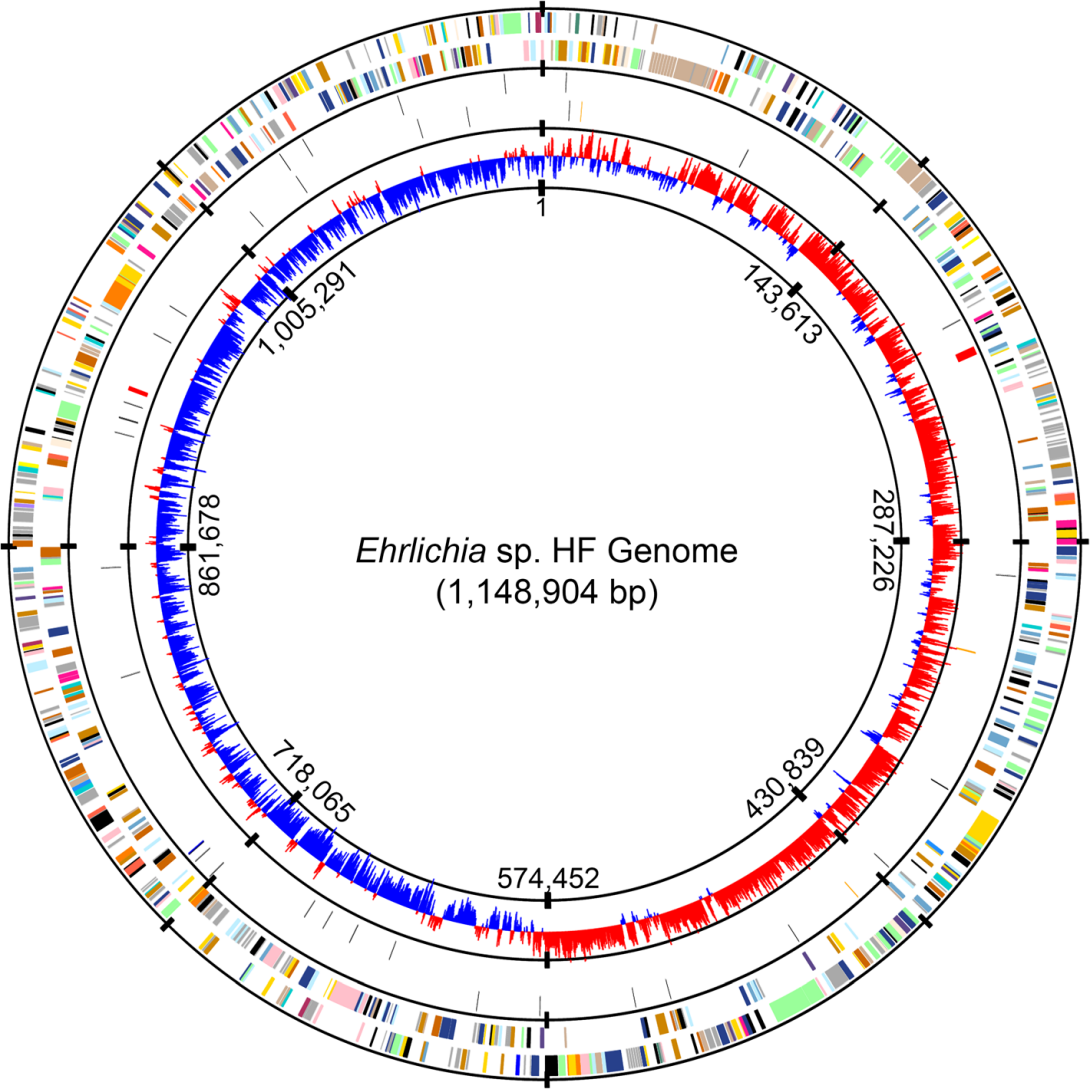
Circular representation of *Ehrlichia* sp. HF genome. From outside to inside, the first circle represents predicted protein coding sequences (ORFs) on the plus and minus strands, respectively. The second circle represent RNA genes, including tRNAs (black), rRNAs (red), tmRNAs (blue), and ncRNAs (orange). The third circle represents GC skew values [(G-C)/(G+C)] with a windows size of 500 bp and a step size of 250 bp. Colors indicate the functional role categories of ORFs - black: hypothetical proteins or proteins with unknown functions; gold: amino acid and protein biosynthesis; sky blue: purines, pyrimidines, nucleosides, and nucleotides; cyan: fatty acid and phospholipid metabolism; light blue: biosynthesis of cofactors, prosthetic groups, and carriers; aquamarine: central intermediary metabolism; royal blue: energy metabolism; pink: transport and binding proteins; dark orange: DNA metabolism and transcription; pale green: protein fate; tomato: regulatory functions and signal transduction; peach puff: cell envelope; pink: cellular processes; maroon: mobile and extrachromosomal element functions

The complete genome of *Ehrlichia* sp. HF was sequenced using both Illumina and PacBio platforms, and the reads from both platforms were combined at multiple levels in order to obtain a reliable assembly. The genome was rotated to the replication origin of *Ehrlichia* sp. HF (Fig. 1), which was predicted to be the region between *hemE* (uroporphyrinogen decarboxylase, EHF_0001) and *tlyC* (hemolysin or related HlyC/CorC family transporter, EHF_0999) as described for other members in the family Anaplasmataceae [70]. Annotation of the finalized genome assembly was generated using the IGS prokaryotic annotation pipeline [71]. The completed genome of *Ehrlichia* sp. HF is a single double-stranded circular chromosome of 1,148,904 bp with an overall G+C content of ~30%, which is similar to those of *E. chaffeensis* Arkansas [72], *E. muris* subsp. *eauclairensis* Wisconsin [19], and *E. muris* AS145^T^ [73] (Table 2).

**Table 2:** Genome properties of representative *Ehrlichia* species

*Ehrlichia* Species^1^	EHF	ECH	EMU	EmCRT^2^	ECA	ERW
NCBI RefSeq	NZ_CP007474	NC_007799	NC_023063	NZ_LANU01000001	NC_007354	NC_005295
Size (bp)	1,148,904	1,176,248	1,196,717	1,148,958	1,315,030	1,516,355
GC (%)	29.6	30.1	29.7	29.8	29.0	27.5
Protein	**866**	**892**	**874**	**866**	**933**	**934**
tRNA	36	37	37	36	36	36
rRNA	3	3	3	3	3	3
Other RNA	4	3	3	4	3	4
Pseudogene	11	17	24	15	10	18
Total Gene	**920**	**952**	**941**	**924**	**985**	**995**

^1^*Abbreviations*: *EHF* *Ehrlichia* sp. HF (HF565), *EMU* *E. muris* subsp. *muris* AS145, *EmCRT* *E. muris* subsp. *eauclairensis* Wisconsin, *ECH* *E. chaffeensis* Arkansas, *ECA* *E. canis* Jake, *ERW* *E. ruminantium* Welgevonden

^2^The genome of *E. muris* subsp. *eauclairensis* Wisconsin is incomplete, consisting of 3 contigs, NZ_LANU01000001, NZ_LANU01000002, and NZ_LANU01000003

The *Ehrlichia* sp. HF genome encodes one copy each of the 5S, 16S, and 23S rRNA genes, which are separated in 2 locations with the 5S and 23S rRNA being adjacent (Fig. 1, red bars in the middle circle) as in other sequenced members in the family Anaplasmataceae [72, 74]. Thirty-six tRNA genes are identified with cognates for all 20 amino acids (AA) (Table 2 and Fig. 1, black bars in the middle circle), similar to other *Ehrlichia* spp. (36 – 37 genes, Table 2).

**Comparative genomic analysis of** ***Ehrlichia*** **sp. HF with other** ***Ehrlichia*** **species**

**Fig. 2:**
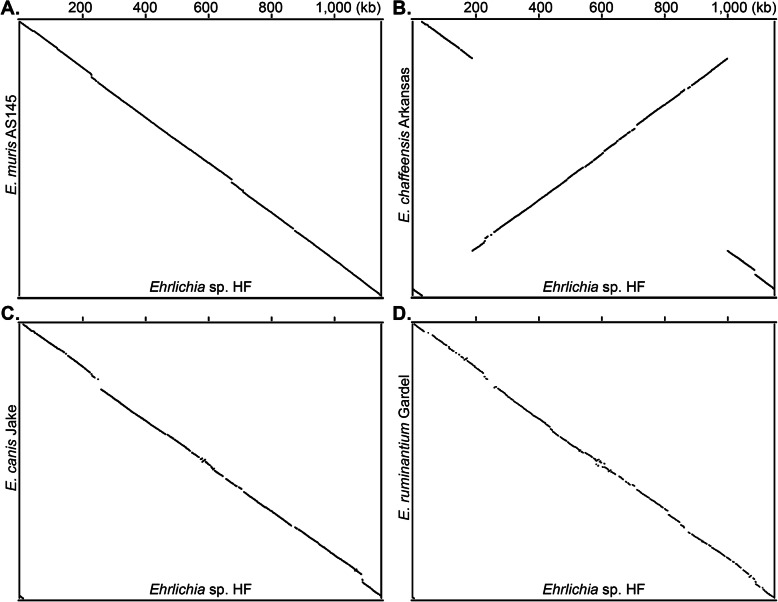
Whole genome alignment between *Ehrlichia* sp. HF and three *Ehrlichia* spp. Genome sequences were aligned between *Ehrlichia* sp. HF and *E. muris* subsp. *muris* AS145 **a**, *E. chaffeensis* Arkansas **b**, *E. canis* Jake **c**, or *E. ruminantium* Gardel **d** using MUGSY program with default parameters, and the graphs were generated using GMAJ. *Ehrlichia* sp. HF genome has a single large-scale symmetrical inversion with *E. chaffeensis*, but exhibits almost complete synteny with other *Ehrlichia* spp

Previous studies have shown that some *Anaplasma* spp. and *Ehrlichia* spp. have a single large-scale symmetrical inversion (X-alignment) near the replication origin, which may have resulted from recombination between duplicated, but not identical *rho* termination factors [72, 75, 76]. All genomes of the sequenced *Ehrlichia* spp. encode duplicated *rho* genes. Whole genome alignments demonstrate that the *Ehrlichia* sp. HF genome exhibits almost complete synteny with other *Ehrlichia* spp., including *E. muris*, *E. canis*, and *E. ruminantium*, without any significant genomic rearrangements or inversions despite these genomes being oriented in the opposite directions (Fig. 2). However, *Ehrlichia* sp. HF has a single large-scale symmetrical inversion relative to *E. chaffeensis* at the duplicated *rho* genes (Fig. 2b). Large scale inversion was also reported in other bacteria such as *Yersinia* and *Legionella* species when genomes of closely related species are compared [77]. However, the biological meaning and evolutionary implications of such process, if any, are largely unknown.

**Fig. 3:**
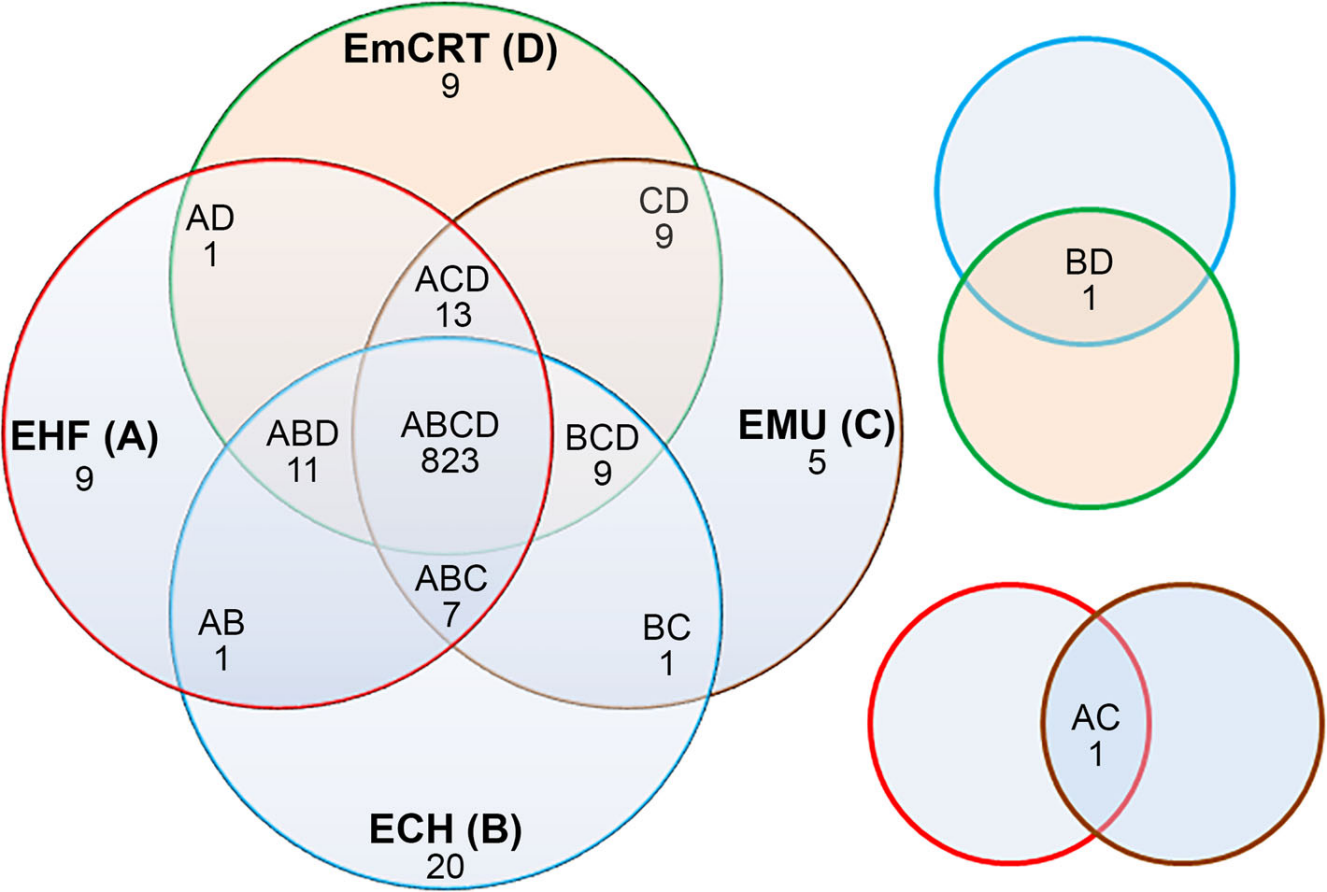
Numbers of protein homologs conserved among representative *Ehrlichia* spp. A Venn diagram was constructed showing the comparison of conserved and unique genes between *Ehrlichia* spp. as determined by reciprocal Blastp algorithm using an E-value of < 1e^-10^. Numbers within the intersections of different circles indicate protein homologs conserved within 2, 3, or 4 organisms. Species indicated in the diagram are abbreviated as follows: EHF **a**, *Ehrlichia* sp. HF; ECH **b**, *E. chaffeensis* Arkansas; EMU **c**, *E. muris* subsp. *muris* AS145; EmCRT **d**, *E. muris* subsp. *eauclairensis* Wisconsin.

In order to compare the protein ortholog groups among four closely-related *Ehrlichia* spp., including *Ehrlichia* sp. HF, *E. muris* subsp. *eauclairensis*, *E. muris* AS145, and *E. chaffeensis* Arkansas, 4-way comparisons were performed using reciprocal Blastp algorithm with E-value < 1e^-10^ (Fig. 3). The four-way comparison showed that the core proteome, defined as the set of proteins present in all four genomes, consists of 823 proteins representing 94.9% of the total 867 protein-coding ORFs in *Ehrlichia* sp. HF (Fig. 3 and Table 3). Among these conserved proteins, the majority are associated with housekeeping functions and are likely essential for *Ehrlichia* survival (Table 3).

**Table 3:** Role category breakdown of protein coding genes in *Ehrlichia* species

Role Category^1^	EHF	ECH	EMU	EmCRT	Unique in EHF^2^
Amino acid biosynthesis	22	23	23	22	
Biosynthesis of cofactors, prosthetic groups, and carriers	64	60	65	61	
Cell envelope	53	51	51	48	1
Cellular processes	42	41	42	41	
Central intermediary metabolism	3	3	5	3	
DNA metabolism	41	44	41	42	
Energy metabolism	84	82	80	83	
Fatty acid and phospholipid metabolism	20	19	21	21	
Mobile elements	4	4	4	4	
Protein fate	79	78	77	78	
Protein synthesis	108	108	107	107	
Nucleotide biosynthesis	35	35	35	35	
Regulatory functions	14	15	13	14	
Transcription	21	21	19	19	
Transport and binding proteins	33	33	32	33	
Hypothetical proteins or proteins with unknown functions	244	276	268	255	8
Total Assigned Functions:	**623**	**617**	**615**	**611**	
Total Proteins	**867**	**893**	**883**	**866**	

^1^*Abbreviations*: *EHF Ehrlichia* sp. HF, *ECH E. chaffeensis* Arkansas, *EMU E. muris* subsp. *muris* AS145, *EmCRT E. muris* subsp. *eauclairensis* Wisconsin.

^2^Proteins specific to *Ehrlichia* sp. HF are based on 4-way comparison analysis among four *Ehrlichia* spp. by Blastp (E < 1e-10)

By 4-way comparison, a hypothetical protein (EHF_RS02845 or MR76_RS01735) is found only in *Ehrlichia* sp. HF and *E. muris* subsp. *muris*, the two strains that do not infect humans, but not in *E. chaffeensis* and *E. muris* subsp. *eauclairensis*, which both infect humans [11, 12, 78] (Table S1). On the other hand, the human-infecting strains of *E. chaffeensis* and *E. muris* subsp. *eauclairensis* have genes encoding a bifunctional DNA-formamidopyrimidine glycosylase/DNA-(apurinic or apyrimidinic site) lyase protein, MutM (ECH_RS02515 or EMUCRT_RS01070) (Table S1). In addition, transposon mutagenesis studies have identified intragenic insertions of genes encoding DNA mismatch repair proteins MutS and MutL in *Ehrlichia* sp. HF [56]. Biological relationship between MutM and the human infectivity remains to be investigated.

*E. muris* subsp. *muris*, *E. muris* subsp. *eauclairensis*, and *Ehrlichia* sp. HF cause persistent or lethal infection in mice, whereas immunocompetent mice clear *E. chaffeensis* infection within 10 – 16 days [79–81]. A metallophosphoesterase (ECH_RS03950/ECH_0964), which may function as a phosphodiesterase or serine/threonine phosphoprotein phosphatase, was found only in *E. chaffeensis* but not in the other three *Ehrlichia* spp. (Table S2).

Except for 28 *E. chaffeensis-*specific proteins, there are less than 10 species-specific proteins present in *Ehrlichia* sp. HF, *E. muris* subsp. *muris* AS145, or *E. muris* subsp. *eauclairensis* (Table S2), all of which are hypothetical proteins without any known functions or domains. Potentially, these proteins may be involved in differential pathogenesis of these *Ehrlichia* species.

Two-way comparisons identified further proteins that are unique to *Ehrlichia* sp. HF, but absent in other *Ehrlichia* spp. (Table S3). Several of these proteins are involved in DNA metabolism, mutation repairs, or regulatory functions that were only found in *Ehrlichia* sp. HF (Table S3). For example, compared to *Ehrlichia* sp. HF proteomes, *E. chaffeensis* lacks a patatin-like phospholipase family protein (ECH_RS03820, a pseudogene with internal frameshift at AA^180^), which has phospholipase A_2_ activity catalyzing the nonspecific hydrolysis of phospholipids, glycolipids, and other lipid acyl hydrolase activities [82–84]. *E. muris* subsp. *muris* lacks CckA protein, a histidine kinase that can phosphorylate response regulator CtrA and regulate the DNA segregation and cell division of *E. chaffeensis* [85, 86]. However, the absence of these proteins needs to be further validated since sequencing errors and mis-annotations can frequently confound such analyses. For example, although the homolog to *E. chaffeensis* TRP120 was not identified in *E. muris* subsp*. eauclairensis*, Tblastn searches indicated that this ORF is split into two pseudogenes (EMUCRT_0995 and EMUCRT_0731) in two separate contigs of the draft genome sequences. In addition, RpoB/C were misannotated in *E. muris* subsp. *eauclairensis* genome as a concatenated pseudogene EMUCRT_RS04655, whereas several genes encoding GyrA, PolI, AtpG, and CckA of *E. muris* AS145 were annotated as pseudogenes due to frameshifts in homopolymeric tracts (Table S3).

### Metabolic and Biosynthetic Potential

The metabolic potential of *Ehrlichia* sp. HF (Table 3) was analyzed by functional role categories using Genome Properties [87], Kyoto Encyclopedia of Genes and Genomes (KEGG) [88], and Biocyc [89]. In addition, by two and four-way comparisons between *Ehrlichia* sp. HF and *E. chaffeensis* (Fig. 3 and Table 3), results indicated that *Ehrlichia* sp. HF possesses similar metabolic pathways as previously described for *E. chaffeensis* [72]. *Ehrlichia* sp. HF genome encodes pathways for aerobic respiration to produce ATP, including pyruvate metabolism, the tricarboxylic acid (TCA) cycle, and the electron transport chain, but lacks critical enzymes for glycolysis and gluconeogenesis. Similar to *E. chaffeensis*, *Ehrlichia* sp. HF can synthesize fatty acids, nucleotides, and cofactors, but has very limited capabilities for amino acid biosynthesis, and is predicted to make only glycine, glutamine, glutamate, aspartate, arginine, and lysine. *Ehrlichia* sp. HF encodes very few enzymes related to central intermediary metabolism (Table 3) and partially lacks genes for glycerophospholipid biosynthesis, rendering this bacterium dependent on the host for its nutritional needs, like *E. chaffeensis* [90, 91].

*Ehrlichia* species, including the HF strain and *E. chaffeensis*, are deficient in biosynthesis pathways of typical pathogen-associate molecular patterns (PAMPs), including lipopolysaccharide, peptidoglycan, common pili, and flagella. Nevertheless, both *E. chaffeensis* and *Ehrlichia* sp. HF induce acute and/or chronic inflammatory cytokines production in a MyD88-dependent, but Toll-like receptors (TLR)-independent manner [92–94]. Similar to acute severe cases of HME, *Ehrlichia* sp. HF causes an acute toxic shock-like syndrome in mice involving many inflammatory factors and kills mice in 10 days [56, 61, 66, 67], suggesting that *Ehrlichia* species have unique, yet to be identified inflammatory molecules.

### Two-component regulatory systems

A two-component regulatory system (TCS) is a bacterial signal transduction system, generally composed of a sensor histidine kinase and a cognate response regulator, which allows bacteria to sense and respond rapidly to environmental changes [95]. Our previous studies showed that *E. chaffeensis* encodes three pairs of TCSs, including CckA/CtrA, PleC/PleD, and NtrX/NtrY, and that the histidine kinase activities were required for bacterial infection [85, 86]. Analysis showed that all three histidine kinases were identified in four species of *Ehrlichia* including *Ehrlichia* sp. HF (Table 4). However, the response regulator *cckA* gene of *E. muris* subsp. *muris* AS145 was annotated as a pseudogene due to an internal frameshift (Table 4). Since CckA regulates the critical biphasic developmental cycle of *Ehrlichia*, which converts between infectious compact dense-cored cell (DC) and replicative larger reticulate cell (RC) form [85], the mutation of *cckA* in *E. muris* AS145 needs to be further validated to rule out sequencing error in a homopolymeric tract.

**Table 4:** Potential pathogenic genes in *Ehrlichia* sp. HF, *E. chaffeensis*, *E. muris* subsp. *muris*, and *E. muris* subsp. *eauclairensis*

Organisms^**1**^	EHF	ECH	EMU	EmCRT
**Outer Membrane Proteins:**				
Omp-1/P28 family proteins	23	22	20	20
Omp85	+	+	+	+
OmpH	+	+	+	+
OmpA family protein	+	+	+	+
EtpE	+	+	+	+
**Type IV Secretion System:**				
VirB1/B5	-	-	-	-
VirB2	+ (5)	+ (4)	+ (4)	+ (5)
VirB3	+	+	+	+
VirB4	+ (2)	+ (2)	+ (2)	+ (2)
VirB6	+ (4)	+ (4)	+ (4)	+ (4)
VirB7	+	+	+	+
VirB8	+ (2)	+ (2)	+ (2)	+ (2)
VirB9	+ (2)	+ (2)	+ (2)	+ (2)
VirB10/B11/D4	+	+	+	+
**Putative T4SS Effectors:**				
Etf-1	+	+	+	+
Etf-2^2^	±	+	±	±
Etf-3	+	+	+	+
**Type I Secretion System**^**3**^	+	+	+	+
**Twin-arginine Translocation (TAT) Pathway**^**4**^	+	+	+	+
**TRP Proteins**				
TRP32	+ (94 aa)	+ (198 aa)	+ (112 aa)	+ (105 aa)
TRP47	+ (255 aa)	+ (316 aa)	+ (228 aa)	+ (252 aa)
TRP120	+ (584 aa)	+ (548 aa)	+ (1288 aa)	+^5^
**Ankyrin-repeat domain proteins**	5	5	5	5
**Two-Component Regulatory Systems:**				
PleC/PleD	+	+	+	+
NtrY/NtrX	+	+	+	+
CckA/CtrA	+	+	±^6^	+

^1^Abbreviations: EHF, *Ehrlichia* sp. HF; EMU, *E. muris* subsp. *muris* AS145; ECH, *E. chaffeensis* Arkansas; EmCRT, *E. muris* subsp. *eauclairensis* Wisconsin. Numbers inside parentheses indicate the copy number of the gene; or else, only a single copy exists. +, genes present; -, homolog of the gene not identified based on Blast searches.

^2^In addition to Etf-2 (ECH_0261, 264 aa), *E. chaffeensis* encodes six paralogs of Etf-2 with protein sizes range from 190 ~ 350 AA (ECH_0243, 293 aa; ECH_0246, 285 aa; ECH_0247, 316 aa; ECH_0253, 189 aa; ECH_0255, 352 aa; and ECH_0257, 226 aa). However, only low homologies (26 ~ 32% AA sequence identity) to *E. chaffeensis* Etf-2 were identified in other Ehrlichia spp. (indicated by ±)

^3^Type I Secretion System is consisting of an outer membrane channel protein TolC, a membrane fusion protein HlyD, and an ATPase HlyB. All are present in these Ehrlichia spp

^4^Both twin-arginine translocase subunits TatA and TatC were identified in all Ehrlichia spp

^5^Tblastn search indicates that that the homolog of *E. chaffeensis* TRP120 in *E. muris* subsp. *eauclairensis* Wisconsin is split into two pseudogenes (EMUCRT_0995 and EMUCRT_0731) present in two separate contigs (NZ_LANU01000002 and NZ_LANU01000003) of the incomplete genome sequences

^6^Gene encoding CtrA protein was identified in *E. muris* subsp. *muris *AS145 genome. However, *cckA* gene is annotated as a pseudogene due to an internal deletion, causing frameshift at 1,123 bp

### *Ehrlichia* Outer Membrane Proteins (Omps)

**Fig. 4:**
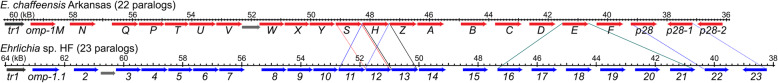
Gene structures of Omp-1/P28 family outer membrane proteins. *E. chaffeensis* Arkansas encodes 22 copies of Omp-1/P28 major outer membrane proteins clustered in tandem. *Ehrlichia* sp. HF encodes 23 copies, which are named Omp-1.1 to Omp-1.23 consecutively. However, it lacks homologs to *E. chaffeensis* Omp-1Z, C, D, F, and P28-2, but has duplicated Omp-1H and 6 copies of Omp-1E (based on best Blastp matches to *E. chaffeensis* Omp-1/P28 proteins). Note: *omp*-*1.1* of *Ehrlichia* sp. HF (EHF_0067, ortholog of *E. chaffeensis omp-1m*) was initially annotated as a pseudogene by NCBI automated annotation pipeline. New start site was determined based on homolog to *E. chaffeensis omp-1m*. Grey bars indicate non-*omp-1* genes within *Ehrlichia* *omp-1/p28* gene clusters

*Ehrlichia* spp. encode 14 – 23 tandemly-arrayed paralogous Omp-1/P28 major outer membrane family proteins in a >26 kb genomic region [52, 93, 96–98]. This polymorphic multigene family is located downstream of *tr1*, a putative transcription factor, and upstream of *secA* gene [97]. Compensating for incomplete metabolic pathways, the major outer membrane proteins P28 and Omp-1F of *E. chaffeensis* possess porin activities for nutrient uptake from the host, which allow the passive diffusion of l-glutamine, the monosaccharides arabinose and glucose, the disaccharide sucrose, and even the tetrasaccharide stachyose as determined by a proteoliposome swelling assay [99]. The *Ehrlichia* sp. HF genome has 23 paralogous *omp-1/p28* family genes, named *omp-1.1* to *omp-1.23* (Fig. 4), and similarly flanked by *tr1* and *secA* genes. Comparing with the *E. chaffeensis* Omp-1/P28 proteins by the best matches from Blastp search, the HF genome lacks orthologs of *E. chaffeensis* Omp-1Z, C, D, F, and P28-2, but has duplicated Omp-1H and 6 copies of Omp-1E (Fig. 4). Since P28 and OMP-1F of *E. chaffeensis* showed different solute diffusion rates [99], the divergence of *Ehrlichia* sp. HF Omp-1 protein family could affect the effectiveness of nutrient acquisition by these bacteria.

Gram-negative bacteria encode a conserved outer membrane protein Omp85 (or YaeT) for outer membrane protein assembly [100, 101], and a molecular chaperone OmpH that interacts with unfolded proteins as they emerge in the periplasm from the Sec translocation machinery [102, 103]. The outer membrane lipoprotein OmpA of *E. chaffeensis* is highly expressed [104–106], and OmpA family proteins in other gram-negative bacteria are well characterized for their roles in porin functions, bacterial pathogenesis, and immunity [107]. All three outer membrane proteins were identified in *Ehrlichia* sp. HF, and highly conserved in these *Ehrlichia* spp. (Table 4), suggesting their essential roles in bacterial infection and survival.

Our previous studies showed that *E. chaffeensis* uses its outer membrane invasin EtpE to bind host cell receptor DNase X, and regulates signaling pathways required for entry and concomitant blockade of reactive oxygen species production for successful infection of host monocytes [108–111]. Analysis showed that the homologs of EtpE were present in *Ehrlichia* sp. HF as well as other *Ehrlichia* (Table 4), suggesting these bacteria might use similar mechanisms for entry and infection of their host cells.

### Protein secretion systems

*Ehrlichia* sp. HF encodes all major components for the Sec-dependent protein export system to secrete proteins across the membranes. In addition, intracellular bacteria often secrete effector molecules into host cells via Sec-independent pathways, which regulate host cell physiological processes, thus enhancing bacterial survival and/or causing diseases [112]. Analysis of the *Ehrlichia* sp. HF genome identifies the Sec-independent Type I secretion system (T1SS), which can transport target proteins with a C-terminal secretion signal across both inner and outer membranes into the extracellular medium, and twin-arginine dependent translocation (TAT) pathway, which can transport folded proteins across the bacterial cytoplasmic membrane by recognizing N-terminal signal peptides harboring a distinctive twin-arginine motif (Table 4) [113].

The Type IV secretion system (T4SS) is a protein secretion system of Gram-negative bacteria that can translocate bacterial effector molecules into host cells and plays a key role in pathogen-host interactions [90, 114]. Except for VirB1 and VirB5, all key components of the T4SS apparatus were identified in *Ehrlichia* sp. HF, similar to those of *E. chaffeensis* (Table 4). The minor pilus subunit VirB5 is absent in all Rickettsiales [115]. VirB1, which is involved in murein degradation, is not present in *Ehrlichia* spp., likely due to the lack of peptidoglycan. These *virB/D* genes encoding T4SS apparatus are split into three major operons as well as single genes in three separate loci that encode VirB7 and duplicated VirB8/9 proteins (Table 4 and Fig. S2). Genes encoding VirB4 are also duplicated, which are clustered with multiple paralogs of *virB2* and *virB6* genes (Table 4 and Fig. S2). *Ehrlichia* sp. HF encodes four tandem functionally uncharacterized VirB6-like paralogs (800 – 1,942 AA), which have increasing masses and are three- to six-fold larger than *Agrobacterium tumefaciens* VirB6 (~300 AA), with extensions found at both N- and C-terminus [116].

**Fig. 5:**
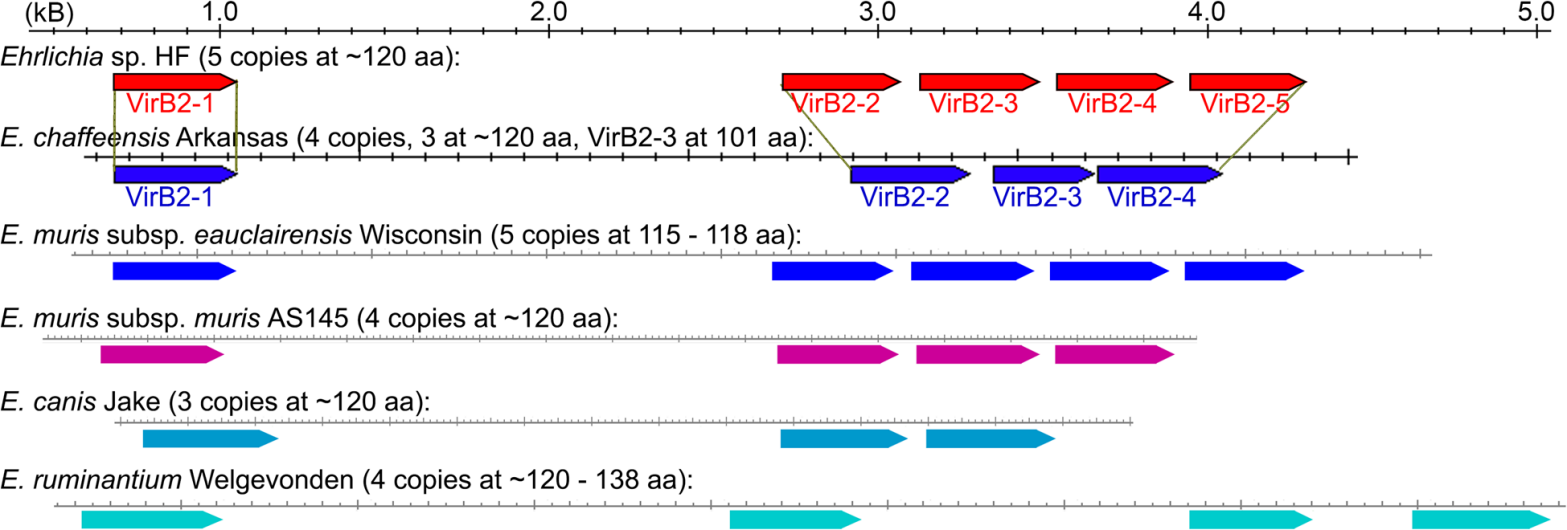
Gene structures of *Ehrlichia* VirB2 paralogs. All *Ehrlichia* spp. encodes 3 - 5 copies of VirB2 paralogs. *Ehrlichia* sp. HF and *E. muris* subsp. *eauclairensis* encode five VirB2 paralogs at ~120 AA, whereas *E. chaffeensis* and *E. muris* subsp. *muris* subsp. *muris* AS145 encode four VirB2. *E. canis* only encodes only 3 copies of VirB2 paralogs, and *E. ruminantium* encodes 4 copies with larger gaps between each *virB2* paralogs. Except for *E. ruminantium*, most *virB2* genes are clustered in tandem with *virB2-1* separated from the rest.

In *A. tumefaciens*, VirB2 is the major T-pilus component that forms the main body of this extracellular structure, which is believed to initiate cell-cell contact with plant cells prior to the initiation of T-complex transfer [117, 118]. A yeast two-hybrid screen identified interaction partners in *Arabidopsis thaliana*, suggesting that *Agrobacterium* VirB2 directly contacts the host cell during the substrate translocation process [114, 119, 120]. Compared to *E. chaffeensis* and *E. muris* subsp. *muris* AS145 that encode four VirB2 paralogs, both *Ehrlichia* sp. HF and *E. muris* subsp. *eauclairensis* encode five VirB-2 paralogs at ~120 AA (Table 4 and Fig. 5). Most *virB2* genes are clustered in tandem except for *virB2-1*, which is separated from the rest. VirB2 paralogs are quite divergent and only share 26% identities despite their similar sizes and domain architecture among Rickettsiales [115, 121]. Phylogenetic analysis of VirB2 paralogs in representative *Ehrlichia* species showed that VirB2-1 proteins are clustered in a separate branch; whereas the rest of VirB2 paralogs are more divergent (Fig. S3). *A. tumefaciens* VirB2 undergoes a novel head-to-tail cyclization reaction and polymerizes to form the T-pilus [116], and mature VirB2 integrates into the cytoplasmic membrane via two hydrophobic α-helices [122, 123]. Analysis of *Ehrlichia* sp. HF VirB2-4 showed that it possesses a signal peptide (cleavage site between residues 29 and 30) and two hydrophobic transmembrane α-helices (Fig. S4A). Alignment of these VirB2 paralogs showed that two hydrophobic α-helices are completely conserved, although they are more divergent on the N- and C-terminus (Fig. S4B), suggesting that *Ehrlichia* VirB2s could form the secretion channels for mature T4SS pili as in *Agrobacterium* [121]. Our previous study confirmed that VirB2 is expressed on the surface of a closely related bacterium *Neorickettsia risticii* [124]. Studies indicated that VirB2 paralogs of *Anaplasma phagocytophilum* are differentially expressed in tick and mammalian cells [125], and an outer membrane vaccine of *Anaplasma marginale* containing VirB2 can protect against the disease and persistent infection [126, 127]. Therefore, the expression of VirB2 paralogs could be specific to the host environment, and their highly divergent C-terminus may offer antigenic variations for protection from host adaptive immunity.

### Putative T4SS Effectors

In contrast to other intracellular pathogens with enormous numbers of effectors (i.e. *Legionella pneumophila*), *E. chaffeensis* encodes much fewer but versatile effectors [128]. Three *E. chaffeensis* T4SS effectors have been experimentally characterized, namely *Ehrlichia* translocated factor (Etf)-1, -2, and -3 [129]. These T4SS effectors are essential for infection of host cells, through inhibition of host apoptosis by Etf-1 [129], acquisition of host nutrients by Etf-1-induced autophagosomal pathways [90], or maintenance of the bacterial replication compartments by Etf-2-mediated inhibition of endosome maturation [130]. Homologs of Etf-1 and Etf-3 were identified in all *Ehrlichia* spp., and they are highly conserved with percent protein identities over 77% and 85%, respectively. Etf-2 proteins are more divergent among *Ehrlichia* spp., and *E. chaffeensis* encodes five paralogs of Etf-2 with protein lengths range from 190 ~ 350 AA; however, only low homologies (26 ~ 32% protein identity) to *E. chaffeensis* Etf-2 were identified in *Ehrlichia* sp. HF and other *Ehrlichia* species (Table 4). Whether these proteins contain a T4SS motif and can be secreted into the host cell cytoplasm remains to be studied.

### Ankyrin-repeat containing proteins

**Fig. 6:**
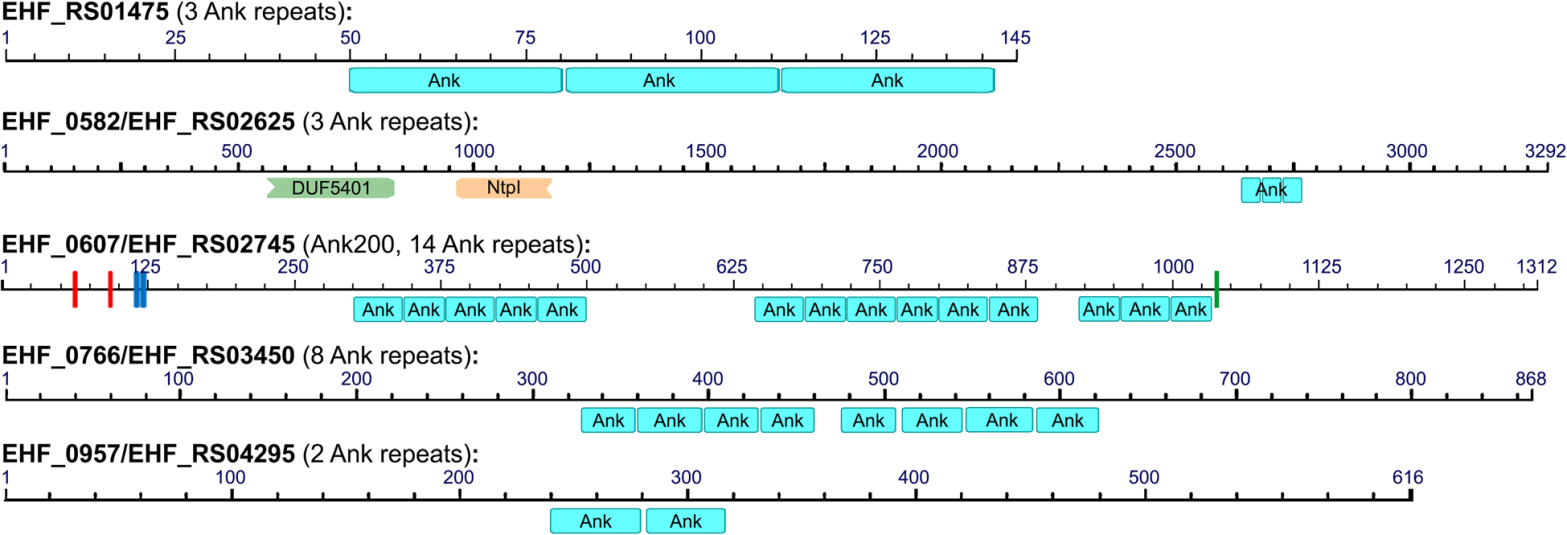
Domain structures of Ankyrin-repeat containing proteins in *Ehrlichia* sp. HF. *Ehrlichia sp.* HF encodes 5 Ank-repeat containing proteins, including *E. chaffeensis* Ank200 homolog (EHF_0607). Ank-repeat domains were determined by NCBI Conserved Domains Database (CDD, https://www.ncbi.nlm.nih.gov/Structure/cdd) [140, 141], and eukaryotic phosphorylation sites were determined by Scansite 4.0 (https://scansite4.mit.edu/) [142]. In addition to 14 Ank repeats, Ank200 (EHF_0607) contains two tyrosine kinase phosphorylation sites (red bars), two SH3 domains (blue), and one Ser/Thr kinase site (green). Domain abbreviations: Ank, Ankyrin repeat; DUF5401, family of unknown function initially found in Chromadorea like *Caenorhabditis elegans*; NtpI, Archaeal/vacuolar-type H^+^-ATPase subunit I/STV1.

Ankyrin-repeats (Ank) are structural repeating motifs that consist of 33-AA with two anti-parallel α-helices connected to the next repeat via a loop region [131]. Ank proteins are more common in eukaryotes, which mediate protein–protein interactions involved in a multitude of host processes including cytoskeletal motility, tumor suppression, and transcriptional regulation [131]. AnkA of *A. phagocytophilum* is one of a few known T4SS effectors, which can be translocated into the host cells, tyrosine-phosphorylated, and plays an important role in facilitating intracellular infection by regulating host signaling pathways [132–134]. *A. phagocytophilum* AnkA can also be translocated to the cell nucleus and bind to transcriptional regulatory regions of the *CYBB* locus to suppress host-cell innate immune response [135, 136]. The AnkA homolog in *E. chaffeensis*, Ank200, also contains tyrosine kinase phosphorylation sites and can be tyrosine-phosphorylated in the infected host cells [137, 138]. *E. chaffeensis* Ank200 interacts with Alu-Sx elements to regulate several genes associated with ehrlichial pathobiology [139]. A homolog of *E. chaffeensis* Ank200 was identified in *Ehrlichia* sp. HF (EHF_0607), which also contains two putative tyrosine kinase phosphorylation sites and SH3 domains in addition to 14 Ank repeats [133] (Fig. 6). Our analysis identified four additional Ank-repeat containing proteins in these representative *Ehrlichia* spp. (Table 4). In *Ehrlichia* sp. HF, these proteins range from ~150 to over 3,000 AA in length and contain 2 - 14 copies of Ank repeats (Fig. 6). It remains to be elucidated if any of the ankyrin repeat-containing proteins in *Ehrlichia* sp. HF can be secreted, and whether these proteins regulate host cell signaling to benefit intracellular ehrlichial infection.

### Tandem-repeat containing proteins (TRPs)

**Fig. 7:**
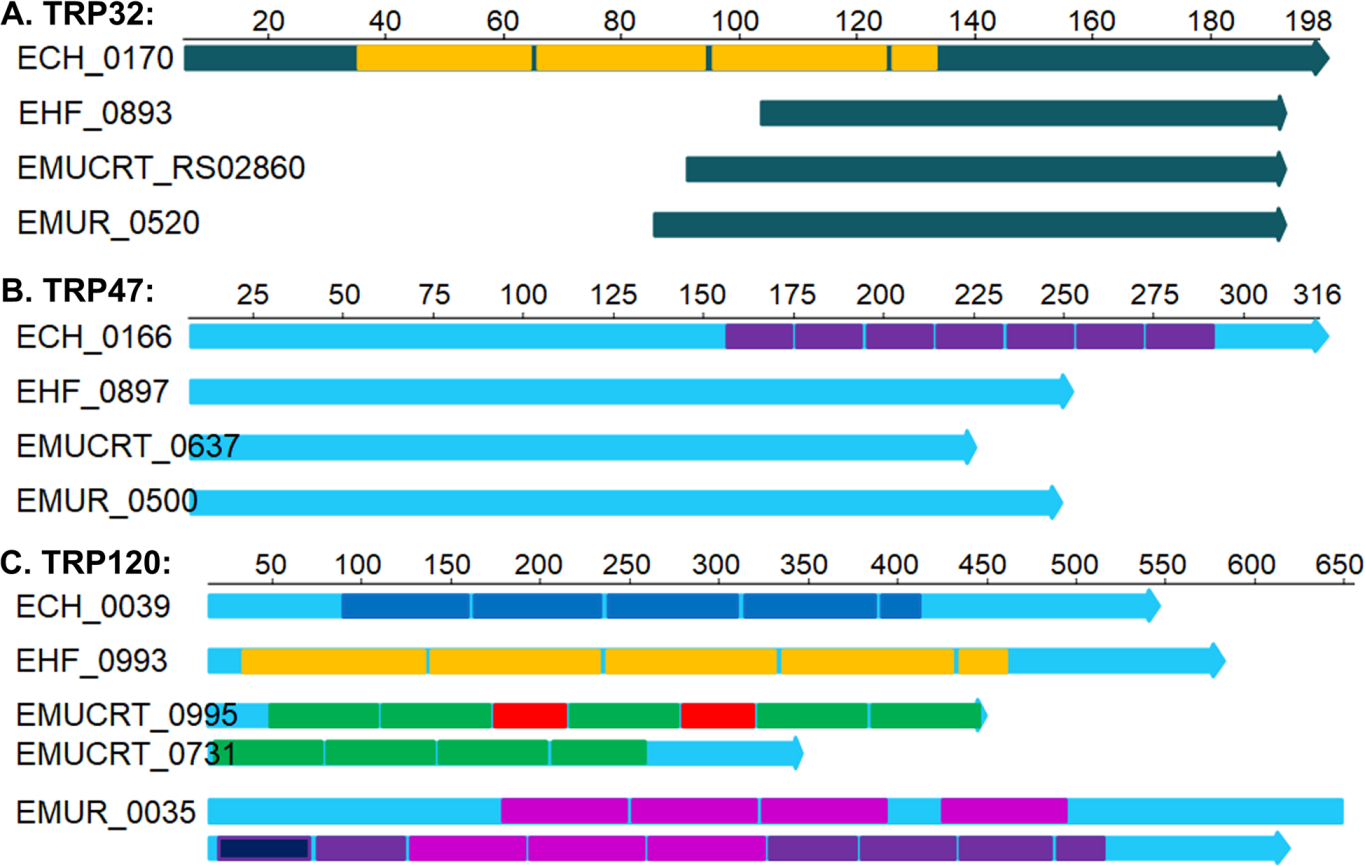
Analysis of *Ehrlichia* tandem repeat proteins TRP-32/47/120. TRP Homologs of *E. chaffeensis* Arkansas were first identified using Blastp among *Ehrlichia* spp., and the internal repeats were determined by XSTREAM (https://amnewmanlab.stanford.edu/xstream/). Colored boxes indicated different repeat sequences and lengths, and were drawn to scale with the protein lengths. TRP proteins are highly variable, and the length and numbers of repeats are different among all *Ehrlichia* spp. **a** *E. chaffeensis* TRP32 (ECH_0170) protein (or variable length PCR target/VLPT, 198 AA) contains 4 consecutive VLPT repeats (30-AA). However, no repeats or VLPT domains were detected in *Ehrlichia* sp*.* HF (EHF_0893/EHF_RS04015, only 90 AA with 45% identity matched to the C-terminus of ECH0170), *E. muris* subsp. *eauclairensis* (EMUCRT_RS02860, 105 AA), and *E. muris* subsp. *muris* (EMUR_00520/MR76_RS00500, 112 AA). **b** *E. chaffeensis* TRP47 protein (ECH_0166, 316 AA) contains eight consecutive 19-AA repeats at its C-terminus. TRP47 homologs in *Ehrlichia* sp. HF (EHF_0897/EHF_RS04625, annotation revised based on Tblastn against *Ehrlichia* sp. HF genome) encodes a smaller protein (255 AA) with 40% identity, mostly conserved in N-terminus. However, no repeat sequences were identified in TRP47 homologs in *Ehrlichia* sp. HF, *E. muris* subsp. *eauclairensis* (EMUCRT_0637/ EMUCRT_RS04575, 252 AA), and *E. muris* subsp. *muris* (EMUR_00500/MR76_RS04630, 228 AA). **c** *E. chaffeensis* TRP120 protein (ECH_0039, 548 AA) contains 4^1/3^ consecutive 80-AA repeats. TRP120 homolog in *Ehrlichia* sp. HF (EHF_0897/EHF_RS04625, 584 AA) contains 4¼ consecutive 100-AA repeats. A much larger protein was identified in *E. muris* subsp. *muris* AS145 (EMUR_0035/MR76_RS00035, 1,288 AA) with 12^1/3^ repeats (8 repeats with 67-AA length and 4^1/3^ repeats of 56-AA length). Two ORFs (EMUCRT_0995 and EMUCRT_09731) in *E. muris* subsp*. eauclairensis* that match to *E. chaffeensis* TRP120 at the N- and C-terminus respectively, were identified in two contigs (NZ_LANU01000002 and NZ_LANU01000003) of the incomplete genome sequences. Nine repeats of 65-AA length were identified in both proteins, whereas two shorter repeats of 38-AA length were found in EMUCRT_0995 only.

Using a heterologous *Escherichia coli* T1SS apparatus, studies have identified four potential *E. chaffeensis* T1SS effectors, including ankyrin-repeat containing protein Ank200, and three tandem-repeat containing proteins (TRPs), TRP47, TRP120, and TRP32 [138]. TRP120 protein also contains a motif that is rich in glycine and aspartate and relates to the repeats-in-toxins (RTX) family of exoproteins [93, 138, 143]. Our current analysis identified homologs of *E. chaffeensis* TRP proteins in *Ehrlichia* sp. HF and other representative *Ehrlichia* spp. (Table 4). In *E. chaffeensis*, all three TRP proteins contain various numbers of tandem repeats with repeat lengths ranging from 19 ~ 80 AA. However, bioinformatic analysis of TRP homologs in *Ehrlichia* spp. indicated that these proteins are highly variable, and the length and numbers of repeats are different among all *Ehrlichia* spp. (Fig. 7, Table S4). Unlike *E. chaffeensis* TRP32 and TRP47, no repeats or variable-length PCR target (VLPT) domains were detected in homologs of those in *Ehrlichia* sp. HF and other *Ehrlichia* spp. (Fig. 7a - b). Interestingly, TRP120 homolog of *Ehrlichia* sp. HF has tandem repeats with longer length (100-AA), whereas that of *E. muris* AS145 encodes a very large protein at 1,288 AA with over 12 repeats that are highly enriched in glutamic acid (Fig. 7c, Table S4). TRP120 homolog is also identified in *E. muris* subsp. *eauclairensis*, which is split into two ORFs in two separate contigs of the incomplete genome sequences, and has a total of ~11 repeats (Fig. 7c, Table S4). Previous studies have indicated that *E. chaffeensis* TRP proteins are highly immunogenic in infected patients and animals [144], and could play important roles in host–pathogen interactions [143, 145–152]. Our recent study using Himar1 transposon mutagenesis of *Ehrlichia* sp. HF recovered a mutant with insertion within *TRP120* gene from DH82 cells, indicating that TRP120 is not essential for survival and infection of *Ehrlichia* sp. HF in DH82 cells [56]. As targeted mutagenesis of *Ehrlichia* is still unavailable, future studies using the cloned TRP120 mutant will benefit functional analysis of TRP120. In addition, it remains to be studied if any of TRPs of *Ehrlichia* sp. HF can be secreted by the T1SS, and whether these proteins regulate host cell signaling to benefit intracellular ehrlichial infection or pathogenicity.

### *Ehrlichia* sp. HF is a new *Ehrlichia* species based on genome and proteome phylogenetic analysis

To classify *Ehrlichia* sp. HF in the genus *Ehrlichia*, we conducted phylogenetic analyses of *Ehrlichia* sp. HF by using nucleotide-based core genome alignment of *Ehrlichia* sp. HF and 6 representative *Ehrlichia* species, subspecies, and strains by using three different parameters: (1) average nucleotide identity (ANI) [153], (2) digital DNA-DNA hybridization (dDDH) [154], and (3) core genome alignment sequence identity (CGASI) [155]. ANI values are calculated by first splitting the genome of one organism into 1 kbp fragments, which are then searched against the genome of the other organism. ANI is then calculated by taking the average sequence identity of all matches spanning >70% of their length with >60% sequence identity [153]. dDDH values are calculated by using the sequence similarity of conserved regions between two genomes and taking the sum of all identities found in matches divided by the overall match length [154]. CGASI values between genomes are calculated by generating a core genome alignment, consisting of all positions present in all analyzed genomes, and calculating the sequence identities between them [155].

**Fig. 8:**
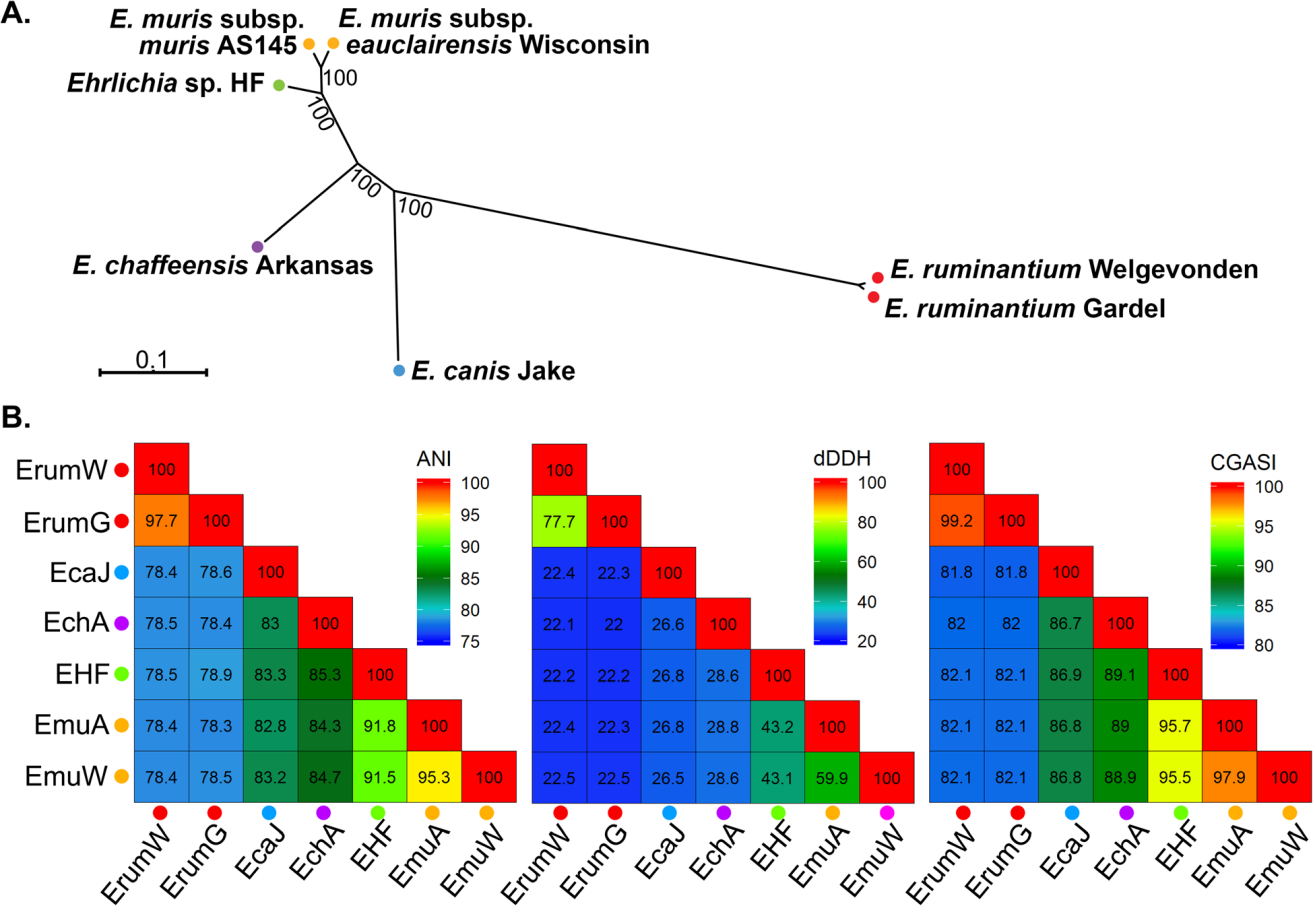
Phylogenetic analysis and determination of ANI, dDDH, and CGASI values of 7 representative *Ehrlichia* species. **a** A maximum-likelihood phylogenetic tree with 1,000 bootstraps was generating using the core genome alignment used to calculate CGASI values. Bootstrap values are indicated next to their respective nodes. **b** The values of ANI, dDDH, and CGASI are calculated between 7 *Ehrlichia* genomes and plotted as a heatmap. The respective values for each pairwise comparison are shown in each cell. Colored circles next to each strain name indicate whether each genome belongs to the same species, with circles of the same color indicating genomes are of the same species according to either ANI, dDDH, or CGASI below the species cutoffs of 95%, 70%, and 96.8%, respectively. Abbreviations and GenBank Accession numbers: EHF, *Ehrlichia* sp. HF (NZ_CP007474.1); EchA, *E. chaffeensis* Arkansas (NC_007799.1); EmuA, *E. muris* subsp. *muris* AS145 (NC_023063.1); EmuW, *E. muris* subsp. *eauclairensis* Wisconsin (NZ_LANU01000001, NZ_LANU01000002, and NZ_LANU01000003); EcaJ, *E. canis* Jake (NC_007354.1); EruW, *E. ruminantium* Welgevonden (NC_005295.2); EruG, *E. ruminantium* Gardel (NC_006831.1).

Using the core genome alignment used to calculate CGASI values, the maximum-likelihood phylogenetic tree of the seven recognized species in the genus *Ehrlichia* showed that *Ehrlichia* sp. HF is a sister taxon to *E. muris* (Fig. 8a), being most closely related to *E. muris* subsp. *muris* AS145. However, between the two genomes, ANI, dDDH, and CGASI values are 91.8%, 43.2%, and 95.7%, respectively, all below the species cutoffs (95%, 70%, and 96.8%, respectively) [155]. Additionally, the current species designations for these 7 *Ehrlichia* genomes are supported by all three parameters, with the exception of two subspecies in *E. muris* (Fig. 8b).

Similar results are observed in a phylogenetic analyses based on the 16S rRNA sequences (Fig. S5A) or eight concatenated protein sequences (3,188 AA total) consisting of five conserved housekeeping proteins (TyrB/Mdh/Adk/FumC/GroEL) and three more divergent surface proteins like major outer membrane or T4SS apparatus proteins (P28/VirB2-1/VirB6-1) (Fig. S5B). However, the nodes on the phylogenetic tree generated using the core nucleotide alignment consistently have higher bootstrap support values than those of 16S rRNAs or concatenated proteins (Fig. 8a and S5). Based on these analyses, we proposed the following new classification of *Ehrlichia* sp. HF.

### Description of *Ehrlichia japonica* sp. nov. (japonica, N.L. fem. adj. *japonica* from Japan)

The distances observed between *Ehrlichia* sp. HF and other *Ehrlichia* species by whole genome sequence-based phylogenetic analysis indicate that *Ehrlichia* sp. HF represents a new species in the genus *Ehrlichia*. This species is therefore named as *Ehrlichia japonica* sp. nov. to denote the geographic region where this bacterium was initially isolated. The type strain, HF^T^, was named after the scientist Hiromi Fujita who first discovered and isolated this bacterium [5].

To date all *E. japonica* was found in various *Ixodes* species of ticks in Japan, France, Serbia, and Romania. This species is highly pathogenic to mice. *E. japonica* can be distinguished by PCR of 16S RNA using *Ehrlichia* sp. HF-specific primer pair HF51f/HF954r (923 bp target size, Table S5, Fig. S6) from other *Ehrlichia* species [156]. *E. japonica* HF^T^ can be stably cultured in DH82 cells, which is available from BEI Resources (Deposit ID# NR-46450, Manassas, VA) and Collection de Souches de l’Unité des Rickettsies (CSUR Q1926, Marseille, France).

## Conclusions

By comparing with closely related *Ehrlichia* spp., this study indicates that the genome of *Ehrlichia* sp. HF encodes all homologs to virulence factors of *E. chaffeensis* required to infect host cells, including outer membrane proteins, protein secretion systems and effectors, supporting that this species can serve as a model bacteria to study in vivo pathogenesis and immune responses for fatal ehrlichiosis. Whole genome alignment and phylogenetic analyses indicate that *Ehrlichia* sp. HF can be classified as a new species in the genus *Ehrlichia*, and we propose to name it as *Ehrlichia japonica* sp. nov. Availability of this bacterial strain in macrophage cultures and complete whole genome sequence data will greatly advance ehrlichiosis researches, including *in vivo* virulence factors, therapeutic interventions, and vaccine studies.

## Methods

### Culture isolation of *Ehrlichia* sp. HF

Two C57BL/6 mice (Envigo, Indianapolis, IN) were intraperitoneally inoculated with mouse spleen homogenates containing *Ehrlichia* sp. HF in RPMI-1640 (Mediatech, Manassas, VA) freezing medium containing 20% fetal bovine serum (FBS; Atlanta Biologicals, Lawrenceville, GA) and 10% DMSO (Millipore Sigma, Burlington, MA), which are stored in liquid nitrogen at approximate 0.35 ml, equivalent to ½ of an infected spleen. Clinical signs and body weight were monitored daily. Moribund mice at 8 day post inoculation were euthanized by CO_2_ inhalation and cervical dislocation. Blood samples were collected by cardiac puncture, and buffy coat was separated by centrifugation at 1,000 × *g*. The presence of *Ehrlichia* sp. HF in monocytes in the blood smear was confirmed by Diff-Quik staining (Thermo Fisher Scientific, Waltham, MA). The spleen was aseptically excised and a single-cell suspension was prepared in 0.7-ml of RPMI-1640 media after lysing red blood cells with ammonium chloride. DH82 cells were cultured in DMEM (Dulbecco minimal essential medium; Mediatech) supplemented with 5% FBS and 2 mM l-glutamine (l-Gln; GIBCO, Waltham, MA) at 37°C under 5% CO_2_ in a humidified atmosphere as described previously [157]. RF/6A cells (ATCC) were cultured in advanced minimal essential medium (AMEM, Gibco) supplemented with 5% FBS and 2 mM l-glutamine. The ISE6 cell line, derived from the *Ixodes scapularis* tick embryo, was cultured in L15C300 medium at 34°C as described previously [158]. Half of buffy coat cells and spleen cell suspension from one mouse were overlaid on DH82 and RF/6A cells in respective culture media, and cultured with the addition of 0.1 μg/mL cycloheximide (Millipore Sigma). To assess the degree of *Ehrlichia* infection in host cells, a drop of infected cells was centrifuged onto a slide in a Shandon Cytospin 4 cytocentrifuge (Thermo Fisher), and the presence of *Ehrlichia-*containing inclusions was examined in both cell types by Diff-Quik staining every 3 – 4 days. *Ehrlichia* sp. HF was continuously passaged in DH82 cells with the addition of 0.1 μg/mL cycloheximide.

### Culture and purification of host cell-free *Ehrlichia* sp. HF and bacterial genomic DNA

Twelve T175 flasks of *Ehrlichia* sp. HF-infected DH82 cells (>80% infectivity) at 3 d post infection (pi) were homogenized in 30 ml of 1× SPK buffer (0.2 M sucrose and 0.05 M potassium phosphate, pH 7.4) for 30 times with type A tight-fitting pestle in a dounce homogenizer (Wheaton, Millville, NJ). After centrifugation at 700 × *g* (Sorvall 6000D, Thermo Fisher), the pellet was further homogenized for additional 30 times. Homogenates were combined and step-wise centrifuged at 700, 1,000, and 1,500 × *g* for 10 min without using the break function of the centrifuge to avoid disturbing the loosely-packed pellets, then passed through 5.0- and 2.7-μm filters, and centrifuged at 10,000 × *g* for 10 min (Sorvall RC 5C Plus using SS-34 rotor). The purity of bacteria was determined by Diff-Quik staining (Fig. S6A). Genomic DNA samples were prepared using Qiagen genomic tips (Qiagen, Germantown, MD) according to the manufacturer’s instructions, and resuspended in TE buffer. The quantity and quality of genomic DNA were determined by Nanodrop (8.41 μg total DNA; Thermo Fisher) as well as 0.9% agarose gel electrophoresis with BioLine markers (Fig. S6B). The purity of bacterial genomic DNA was confirmed by PCR and agarose gel electrophoresis using specific primers targeting *Ehrlichia* sp. HF 16S rRNA gene (HF51f/HF954r) and canine G3PDH DNA (Table S5 and Fig. S6) [156, 159]. The contamination of host DNA was estimated to be satisfactorily low for shotgun sequencing to obtain complete genome sequence (Fig. S6B).

### Sequencing and annotation

Indexed Illumina mate pair libraries were prepared following the mate pair library v2 sample preparation guide (Illumina, San Diego, CA), with two modifications. First, the shearing was performed with the Covaris E210 (Covaris, Wobad, MA) using the following conditions: duty cycle, 10; time, 120 sec; intensity 4; and cycles per burst, 200. The DNA was purified between enzymatic reactions and the size selection of the library was performed with AMPure XT beads (Beckman Coulter Genomics, Danvers, MA).

Paired-end genomic DNA libraries for sequencing using Illumina platform were constructed using the KAPA library preparation kit (Kapa Biosystems, Woburn, MA). DNA was fragmented with the Covaris E210 and the libraries were prepared using a modified version of manufacturer’s protocol. The DNA was purified between enzymatic reactions and the size selection of the library was performed with AMPure XT beads (Beckman Coulter Genomics), using 33.3 μl beads for 50 μl purified ligation product. For indexed samples, the PCR amplification step was performed with primers containing a six-nucleotide index sequence.

Concentration and fragment size of libraries were determined using the DNA High Sensitivity Assay on the LabChip GX (Perkin Elmer, Waltham, MA) and qPCR using the KAPA Library Quantification Kit (Complete, Universal) (Kapa Biosystems, Woburn, MA). The mate pair libraries were sequenced on an Illumina HiSeq 2500 (Illumina), producing 23.8 M reads (4.8G bases), while the paired-end libraries were sequenced on an Illumina MiSeq (Illumina), producing 1.6 M reads (826.2M bases).

DNA samples for PacBio sequencing were sheared to 8 kbp using the Covaris gTube (Woburn, MA). Sequencing libraries were constructed and prepared for sequencing using the SMRTbell Express Template Prep Kit 2.0 (3kbp - 10kbp) and the DNA/Polymerase Binding Kit 2.0 (Pacific Biosciences. Menlo Park, CA). Libraries were loaded onto v2 SMRT Cells, and sequenced with the DNA Sequencing Kit 2.0 (Pacific Biosciences), producing 81,741 reads (388.8M bases). All sequence reads were deposited at NCBI Sequence Read Archive (SRA, BioProject accession number PRJNA187357).

Five assemblies were generated with various combinations of the data and assembly algorithms: (1) Celera Assembler v7.0 of only PacBio data, (2) Celera Assembler v7.0 of PacBio data with correction using Illumina paired-end data, (3) HGAP assembly of only PacBio data, (4) MaSuRCA 1.9.2 assembly of Illumina paired-end data subsampled to 50× coverage, and (5) MaSuRCA 1.9.2 assembly of Illumina paired-end data subsampled to 80× coverage. The first assembly was the optimal assembly, namely the one generated with Celera Assembler v7.0 with only the PacBio data. The data set was subsampled to ~22× coverage of the longest reads using an 8 Kbp minimum read length cutoff, with the remainder of the reads used for the error correction step. The resulting single-contig assembly totaled ~89.4 Kbp with 41.68% GC-content. The genome was trimmed to remove overlapping sequences, oriented, circularized, and rotated to the predicted origin of replication. Annotation for this finalized genome assembly was generated using the IGS prokaryotic annotation pipeline [71] and deposited in GenBank (accession number NZ_CP007474.1).

### ANI, dDDH, and CGASI calculations

ANI values were calculated using OrthiANIu v1.2 [153] paired with USEARCH v9.2.64 [160] run with default settings. dDDH values calculated using the web service GGDC v2.1 (ggdc.dsmz.de) [161] paired with the BLAST+ alignment tool [162] with default settings. For CGASI calculations, core genome alignments were constructed using Mugsy v1r2.2 [163] and mothur v1.40.4 [164]. CGASI values were calculated from the core genomes using the *PairwiseAlignments* function of the R package Biostrings. A maximum-likelihood phylogenetic tree was generated and plotted using IQTree v1.6.2 [165], with ModelFinder [166] and 100 ultra-fast bootstraps [167], and iTOL v5.5.1 [168], respectively.

### Bioinformatic Analysis

For phylogenetic analysis, 16S rRNA genes from seven representative *Ehrlichia* spp., including *Ehrlichia* sp. HF, *E. chaffeensis* Arkansas, *E. muris* subsp. *muris* AS145, *E. muris* subsp. *eauclairensis* Wisconsin, *E. canis* Jake, *E. ruminantium* Welgevonden, and *E. ruminantium* Gardel, were aligned individually using MegAlign program of DNAStar Lasergene 12 (Madison, WI) with MUSCLE algorithm. Alternatively, eight concatenated proteins [169], including 5 conserved housekeeping proteins (aspartate aminotransferase [TyrB], malate dehydrogenase [Mdh], adenylate kinase [Adk], fumarate hydratase [FumC], and 60 kDa chaperon [GroEL]), and 3 divergent outer membranes proteins (P28/VirB2-1/VirB6-1) from these *Ehrlichia* spp. were aligned individually using MegAlign program with CLUSTAL OMEGA. The evolutionary analyses were inferred by using the Maximum Likelihood method and Tamura-Nei model for 16S rRNA [170] or JTT matrix-based model for concatenated proteins [171], and bootstrap values for 1,000 replicates were obtained in MEGA X software [172]. Initial trees for the heuristic search were obtained automatically by applying Neighbor-Join and BioNJ algorithms to a matrix of pairwise distances estimated using the Maximum Composite Likelihood approach, and then selecting the topology with superior log likelihood value. Phylogenetic trees were drawn to scale with branch lengths shown under each branch, and the highest log likelihood is shown. The percentage of trees in which the associated taxa clustered together is shown above the branches.

The GC-skew was calculated as (C-G)/(C+G) in windows of 500 bp with step size of 250 bp along the chromosome. Whole genome alignments between *Ehrlichia* spp. were generated using Mugsy program with default parameters [163], and the graphs were generated using GMAJ (http://globin.bx.psu.edu/dist/gmaj/).

To determine protein orthologs conserved among *Ehrlichia* spp., and *Ehrlichia* species-specific genes compared to other related organisms, orthologous clusters were determined by using the reciprocal Basic Local Alignment Search Tool (BLAST) algorithm Blastp with an E-value of < 1e^–10^.

Metabolic pathways and transporters were compared across genomes using (1) the Protein homologs generated with reciprocal Blastp, (2) Genome Properties [87], (3) TransportDB [173], (4) Kyoto Encyclopedia of Genes and Genomes (KEGG, http://www.kegg.jp), and (5) Biocyc [174]. Signal peptides and membrane proteins were predicted using the pSort-B algorithm (http://psort.org/psortb/) [175], and lipoproteins were predicted by LipoP 1.0 (http://www.cbs.dtu.dk/services/LipoP) [176].

### NCBI GenBank Accession numbers and abbreviations of bacteria

*Ehrlichia* sp. HF (EHF), NZ_CP007474.1; *E. chaffeensis* Arkansas (ECH), NC_007799.1; *E. muris* subsp. *muris* AS145 (EMU), NC_023063.1; *E. muris* subsp. *eauclairensis* Wisconsin (EmCRT, three contigs), NZ_LANU01000001, NZ_LANU01000002, and NZ_LANU01000003; *E. canis* Jake (ECA), NC_007354.1; *E. ruminantium* Welgevonden (ERU), NC_005295.2.

## Notes

During the review process of the present paper, Wang *et al*. [152] reported (published online on August 3, 2020) that an Himar1 transposon insertion mutant in *TRP120* gene of *E. chaffeensis* was recovered in DH82 cells. This mutant had an initial lag phase but recovered afterwards in DH82 cell culture; however, it could not infect dogs when mixtures of *E. chaffeensis* transposon mutants were inoculated into dogs. Future availability of TRP120 mutants of multiple *Ehrlichia* species will help comparative functional analysis of TRP120.

## Supplementary Information

**Additional file 1: Table S1.** *Ehrlichia* proteins shared in two species by 4-way comparison analysis

**Additional file 2: Table S2.** *Ehrlichia* species-specific proteins by 4-way comparison analysis

**Additional file 3: Table S3.** *Ehrlichia* sp. HF-specific proteins by 2-way comparison analysis

**Additional file 4: Table S4.** Internal Repeats of *Ehrlichia* Tandem Repeat Proteins

**Additional file 5: Table S5.** Primers used in this study

**Additional file 6: Figure S1.** Culture Isolation of *Ehrlichia* sp. HF from infected mouse buffy coat and spleen. (A) Body weight of mice inoculated with mouse spleen homogenates containing *Ehrlichia* sp. HF following days post inoculation. (B) *Ehrlichia* sp. HF (white arrows) in the blood monocytes from buffy coat smear by Diff-Quik staining. (C-D) Large *Ehrlichia*-containing inclusions (white arrows) in DH82 cells at ~ 3 weeks post infection (pi) or RF/6A cells at 2 weeks pi. (E) ISE6 cells were infected with purified host cell-free *Ehrlichia* sp. HF-infected DH82 cells and cultured in L15C300 media at 34°C. Infectivity reached 10% at 3 - 5 d pi with large morulae packed with *Ehrlichia*. Bar, 10 μm.

**Additional file 7: Figure S2.** Gene Structures of *Ehrlichia* sp. HF Type IV Secretion System. *Ehrlichia* sp. HF encodes a Type IV secretion system. These *virB/D* genes are split into three major operons: *virB2/4*, virB3/4/6*virB3/4/6*, *virB8/9/10/11/D4*, and three separate loci: *virB7* and duplicated *virB8-2* and *virB9-2*. *virB2* genes are duplicated into 5 copies, whereas *virB6* into 4 copies. Genes encoding *virB1* and *virB5* are not present in HF genome. Note: Due to the short protein length and low homology, *virB7* was not annotated as an ORF by NCBI automated annotation pipeline. However, by TBLASTN using *A. marginale* VirB7 protein sequence [121] against the entire HF genome sequence, a putative *virB7* gene was identified and manual curated.

**Additional file 8: Figure S3.** Phylogenetic analysis of *Ehrlichia* VirB2 paralogs of representative *Ehrlichia* species. Phylogenic tree of VirB2 paralogs of representative *Ehrlichia* species, including *Ehrlichia* sp. HF (EHF), *E. chaffeensis* Arkansas (EchArk), *E. muris* subsp. *muris* AS145 (Emuris), and *E. muris* subsp. *eauclairensis* Wisconsin (EmCRT). The evolutionary history was inferred by using the Maximum Likelihood method and JTT matrix-based model, and the tree with the highest log likelihood is shown. The tree is drawn to scale with branch lengths measured in the number of substitutions per site (below branches), and the percentage of trees in which the associated taxa clustered together is shown above the branches. Evolutionary analyses were conducted in MEGA X.

**Additional file 9: Figure S4.** Domain structures and alignment of VirB2 paralogs of representative *Ehrlichia* species (A) Domain structures of *Ehrlichia* sp. HF VirB2-4. Analysis of *Ehrlichia* sp. HF VirB2-4 showed that it possesses a signal peptide (cleavage site between residues 29 and 30) and two putative transmembrane motifs. The signal peptide and transmembrane helices (TM) were predicted by SignalP-5.0 Server (http://www.cbs.dtu.dk/services/SignalP/) and TMHMM Server 2.0 (http://www.cbs.dtu.dk/services/TMHMM/), respectively. Hydrophobicity was analyzed by Protean program (DNAStar). (B) Alignment of VirB2 paralogs of representative *Ehrlichia* species showed that although these proteins are more divergent on the N- and C-terminus, they are highly conserved in the central transmembrane motifs or hydrophobic regions (indicated by red boxes). *, conserved among all Ehrlichia VirB2 proteins.

**Additional file 10: Figure S5.** Phylogenetic trees of representative *Ehrlichia* species based on 16S rRNA sequences and concatenated protein sequences. (A) 16S rRNA genes from seven representative *Ehrlichia* spp. were aligned individually using MegAlign (1,514 nucleotides of aligned nucleotides). (B) Eight *Ehrlichia* proteins, including 5 conserved housekeeping proteins (TyrB, Mdh, Adk, FumC, and GroEL) and 3 divergent outer membranes proteins (P28, VirB2-1, and VirB6-1) from these *Ehrlichia* spp. were aligned using MegAlign. The aligned protein sequences were trimmed and concatenated (3,188 AA total). The evolutionary analyses were inferred by using the Maximum Likelihood method and Tamura-Nei model for 16S rRNA, or JTT matrix-based model for concatenated proteins. Bootstrap values for 1,000 replicates were obtained using MEGA X. The trees with the highest log likelihood (-2609.17 for 16S rRNA, and -17382.50 for proteins) were shown, and the percentage of trees in which the associated taxa clustered together in the bootstrap test was shown above each branch. The tree is drawn to scale with branch lengths measured in the number of average nucleotide substitutions per site (shown under each branch). GenBank Accession numbers for seven representative *Ehrlichia* spp.: *Ehrlichia* sp. HF, NZ_CP007474.1; *E. chaffeensis* Arkansas, NC_007799.1; *E. muris* subsp. *muris* AS145, NC_023063.1; *E. muris* subsp. *eauclairensis* Wisconsin, LANU01000000; *E. canis* Jake, NC_007354.1; *E. ruminantium* Welgevonden, NC_005295.2; *E. ruminantium* Gardel, NC_006831.1.

**Additional file 11: Figure S6.** Purification of host cell-free *Ehrlichia* sp. HF and bacterial genomic DNA. (A) Twelve T175 flasks of *Ehrlichia* sp. HF-infected DH82 cells (>80% infectivity) at 3d pi were homogenized in 30 ml of 1× SPK for 30 times with type B tight-fitting pestle. Pellet following centrifugation at 700 × g was homogenized for additional 30 times. Both homogenates were step-wise centrifuged at 700, 1,000, and 1,500 × g, passed through 5.0- and 2.7-μm filters, and centrifuged at 10,000 × g for 10 min. Host cell-free *Ehrlichia* sp. HF was purified with very low host nuclear contamination under Diff-Quik staining. Bar, 10 μm. (B) Genome DNAs of *Ehrlichia* sp. HF (EHF) were purified using Qiagen genomic tips and dissolved in TE buffer. DNAs were resolved using 0.9% agarose with BioLine molecular weight (MW) markers with DNA concentrations of each band showing inside parenthesis. Genomic DNA bands above 20 kB were visible, and the concentration was above 15 ng/μl. PCR reactions were carried out with 35 cycles at 98°C for 30-second, 60°C for 30-second, and 68°C for 1-minute. Primers targeting 16S rRNA gene of *Ehrlichia* sp. HF detected specific bands at 1/100 dilutions, but dog G3PDH primers did not amplify any bands under any dilutions (Dilutions: .1, 1/10, .01, 1/100 dilution; Pos.: positive control using DNA isolated from dog DH82 cells; -, negative control without DNA input).

## Data Availability

The datasets supporting the results of this article are included within the article and supplementary information. The genome sequence has been deposited in GenBank with Accession number NZ_CP007474, *Ehrlichia japonica* strain HF is available from BEI Resources (Deposit ID# NR-46450) and CSUR (Q1926).

## Abbreviations

AA: Amino acid
ANI: Average nucleotide identity
BLAST: Basic local alignment search tool
bp: Base pair
CGASI: Core genome alignment sequence identity
dDDH: Digital DNA-DNA hybridization
Etf: *Ehrlichia* translocated factor
HME: Human monocytic ehrlichiosis
ORF: Open reading frame
pi: Post infection
Omp: Outer membrane protein
T1SS: Type I secretion system
T4SS: Type IV secretion system
TCS: Two-component regulatory system
TRP: Tandem-repeat containing protein
