# Supplementary material for: Comparative Analysis of Genome of Ehrlichia sp. HF, a Model Bacterium to Study Fatal Human Ehrlichiosis
Source: BMC Genomics. 2021 Jan 6;22:11. doi: 10.1186/s12864-020-07309-z (PMC7789307; doi:10.1186/s12864-020-07309-z)
Supplement: Supplementary file 1 — Additional file 1: Table S1. Ehrlichia proteins shared in two species by 4-way comparison analysis [file 12864_2020_7309_MOESM1_ESM.docx]

# Supplementary Table 1. *Ehrlichia* orthologous proteins shared in two species by 4-way comparison analysis ^[[1]](#footnote-1)^

| Locus ID (AA numbers) | Protein Name | Function Role Categories | |
| --- | --- | --- | --- |
| **Only present in *Ehrlichia* sp. HF and *E. muris* subsp*. muris* AS145** | | | |
| EHF_RS02845 (85) | hypothetical protein | Hypothetical proteins | |
| MR76_RS01735 (64) |  |  |  |
|  |  |  |  |
| **Only present in *E. chaffeensis* and *E. muris* subsp. *eauclairensis* ^[[2]](#footnote-2)^** | | | |
| ECH_RS02515 (270) | bifunctional DNA-formamidopyrimidine glycosylase/DNA-(apurinic or apyrimidinic site) lyase [*mutM*] | DNA metabolism / DNA replication, recombination, and repair | |
| EMUCRT_RS01070 (270) |  |  |  |

1. Proteins shared only between *Ehrlichia* sp. HF and *E. muris* subsp. *muris* AS145, or *E. muris* subsp. *eauclairensis* Wisconsin and *E. chaffeensis* Arkansas were determined based on 4-way comparison analysis by Blastp algorithm (E-value < 1e^-10^) among these four *Ehrlichia* spp. Protein lengths in amino acid numbers are listed inside parentheses. [↑](#footnote-ref-1)
2. Tblastn searches indicated that both *Ehrlichia* sp. HF and *E. muris* subsp. *muris* have pseudogenes encoding homologs of *mutM*, which are disrupted by internal frameshift. [↑](#footnote-ref-2)
