# Supplementary material for: Comparative Analysis of Genome of Ehrlichia sp. HF, a Model Bacterium to Study Fatal Human Ehrlichiosis
Source: BMC Genomics. 2021 Jan 6;22:11. doi: 10.1186/s12864-020-07309-z (PMC7789307; doi:10.1186/s12864-020-07309-z)
Supplement: Supplementary file 2 — Additional file 2: Table S2. Ehrlichia species-specific proteins by 4-way comparison analysis [file 12864_2020_7309_MOESM2_ESM.docx]

# Supplementary Table 2. *Ehrlichia* species-specific proteins by 4-way comparison analysis ^[[1]](#footnote-1)^

| Locus ID | Protein Name | Protein Length | Function Role Categories |
| --- | --- | --- | --- |
| ***Ehrlichia* sp. HF (9 proteins)** | | | |
| EHF_RS03890 | putative membrane protein | 72 | Cell envelope |
| EHF_RS00770 | hypothetical protein | 78 | Hypothetical proteins |
| EHF_RS00995 | hypothetical protein | 62 | Hypothetical proteins |
| EHF_RS01025 | hypothetical protein | 131 | Hypothetical proteins |
| EHF_RS01045 | hypothetical protein | 117 | Hypothetical proteins |
| EHF_RS01265 | hypothetical protein | 73 | Hypothetical proteins |
| EHF_RS01860 | hypothetical protein | 67 | Hypothetical proteins |
| EHF_RS04240 | hypothetical protein | 79 | Hypothetical proteins |
| EHF_RS04580 | hypothetical protein | 76 | Hypothetical proteins |
|  |  |  |  |
| ***E. muris* subsp. *eauclairensis* Wisconsin (9 proteins)** | | | |
| EMUCRT_RS00115 | hypothetical protein | 79 | Hypothetical proteins |
| EMUCRT_RS00235 | hypothetical protein | 70 | Hypothetical proteins |
| EMUCRT_RS02480 | hypothetical protein | 66 | Hypothetical proteins |
| EMUCRT_RS02530 | hypothetical protein | 111 | Hypothetical proteins |
| EMUCRT_RS02770 | hypothetical protein | 67 | Hypothetical proteins |
| EMUCRT_RS02780 | hypothetical protein | 79 | Hypothetical proteins |
| EMUCRT_RS04645 | hypothetical protein | 98 | Hypothetical proteins |
| EMUCRT_RS03590 | conserved domain protein | 71 | Hypothetical proteins |
| EMUCRT_RS03945 | hypothetical protein | 66 | Hypothetical proteins |
|  |  |  |  |
| ***E. muris* subsp. *muris* AS145 (5 proteins)** | | | |
| MR76_RS01015 | hypothetical protein | 59 | Hypothetical proteins |
| MR76_RS01715 | hypothetical protein | 59 | Hypothetical proteins |
| MR76_RS02570 | hypothetical protein | 71 | Hypothetical proteins |
| MR76_RS03670 | hypothetical protein | 120 | Hypothetical proteins |
| MR76_RS03865 | hypothetical protein | 60 | Hypothetical proteins |
|  |  |  |  |
| ***E. chaffeensis* Arkansas (28 proteins)** | | | |
| ECH_RS03950 | serine/threonine phosphoprotein phosphatase | 423 | Regulatory functions |
| ECH_RS03860 | conserved domain protein | 1349 | Hypothetical proteins |
| ECH_RS00435 | hypothetical protein | 61 | Hypothetical proteins |
| ECH_RS00440 | hypothetical protein | 63 | Hypothetical proteins |
| ECH_RS00445 | hypothetical protein | 61 | Hypothetical proteins |
| ECH_RS00465 | hypothetical protein | 203 | Hypothetical proteins |
| ECH_RS00475 | hypothetical protein | 202 | Hypothetical proteins |
| ECH_RS00485 | hypothetical protein | 211 | Hypothetical proteins |
| ECH_RS01010 | hypothetical protein | 293 | Hypothetical proteins |
| ECH_RS01025 | hypothetical protein | 70 | Hypothetical proteins |
| ECH_RS01035 | hypothetical protein | 205 | Hypothetical proteins |
| ECH_RS01040 | hypothetical protein | 364 | Hypothetical proteins |
| ECH_RS01050 | hypothetical protein | 189 | Hypothetical proteins |
| ECH_RS01065 | hypothetical protein | 118 | Hypothetical proteins |
| ECH_RS01105 | hypothetical protein | 244 | Hypothetical proteins |
| ECH_RS01130 | hypothetical protein | 179 | Hypothetical proteins |
| ECH_RS01145 | hypothetical protein | 198 | Hypothetical proteins |
| ECH_RS01150 | hypothetical protein | 512 | Hypothetical proteins |
| ECH_RS01605 | hypothetical protein | 105 | Hypothetical proteins |
| ECH_RS02395 | hypothetical protein | 134 | Hypothetical proteins |
| ECH_RS02450 | hypothetical protein | 63 | Hypothetical proteins |
| ECH_RS02510 | hypothetical protein | 374 | Hypothetical proteins |
| ECH_RS02535 | hypothetical protein | 322 | Hypothetical proteins |
| ECH_RS02540 | hypothetical protein | 59 | Hypothetical proteins |
| ECH_RS02545 | hypothetical protein | 301 | Hypothetical proteins |
| ECH_RS02555 | hypothetical protein | 230 | Hypothetical proteins |
| ECH_RS04770 | hypothetical protein | 23 | Hypothetical proteins |
| ECH_RS03170 | hypothetical protein | 79 | Hypothetical proteins |

1. Proteins specific to each *Ehrlichia* sp. are determined based on 4-way comparison analysis by Blastp algorithm (E-value < 1e^-10^) among *Ehrlichia* sp. HF, *E. muris* subsp. *eauclairensis* Wisconsin, *E. muris* subsp. *muris* AS145, and *E. chaffeensis* Arkansas. [↑](#footnote-ref-1)
