# Supplementary material for: Comparative Analysis of Genome of Ehrlichia sp. HF, a Model Bacterium to Study Fatal Human Ehrlichiosis
Source: BMC Genomics. 2021 Jan 6;22:11. doi: 10.1186/s12864-020-07309-z (PMC7789307; doi:10.1186/s12864-020-07309-z)
Supplement: Supplementary file 3 — Additional file 3: Table S3. Ehrlichia sp. HF-specific proteins by 2-way comparison analysis [file 12864_2020_7309_MOESM3_ESM.docx]

# Supplementary Table 3. Proteins that are present only in *Ehrlichia* sp. HF, but absent in other *Ehrlichia* spp. by 2-way comparison analysis ^[[1]](#footnote-1)^

| Locus ID | Protein Name | Protein Length | | | Function Role Categories |
| --- | --- | --- | --- | --- | --- |
| **Against *E. muris* subsp. *eauclairensis* Wisconsin (18 proteins)** | | | | | |
| EHF_RS04475 | 120 kDa immunodominant surface protein (TRP120) ^[[2]](#footnote-2)^ | | 584 | Cellular processes: Pathogenesis | |
| EHF_RS01015 | DNA-3-methyladenine glycosylase (*3mg*) ^[[3]](#footnote-3)^ | | 188 | DNA metabolism: DNA replication, recombination, and repair | |
| EHF_RS03720 | DNA-directed RNA polymerase, beta' subunit (*rpoC*) ^[[4]](#footnote-4)^ | | 1410 | Transcription: DNA-dependent RNA polymerase | |
| EHF_RS03725 | DNA-directed RNA polymerase, beta subunit (*rpoB*) ^4^ | | 1380 | Transcription: DNA-dependent RNA polymerase | |
| EHF_RS04645 | DUF3514 domain-containing protein | | 63 | Hypothetical proteins | |
| EHF_RS00980 | hypothetical protein | | 341 | Hypothetical proteins | |
| EHF_RS02560 | conserved domain protein | | 75 | Hypothetical proteins | |
| EHF_RS02840 | hypothetical protein | | 696 | Hypothetical proteins | |
| EHF_RS02845 | hypothetical protein | | 85 | Hypothetical proteins | |
|  |  | |  |  | |
| **Against *E. muris* subsp. *muris* AS145 (22 proteins)** | | | | | |

| EHF_RS03385 | DNA gyrase, A subunit (*gyrA*) ^[[5]](#footnote-5)^ | 898 | DNA metabolism: DNA replication |
| --- | --- | --- | --- |
| EHF_RS04315 | DNA polymerase I (*polA*) ^5^ | 859 | DNA metabolism: DNA replication |
| EHF_RS01070 | putative cytochrome oxidase assembly protein | 344 | Energy metabolism: Electron transport |
| EHF_RS01240 | pyruvate, phosphate dikinase | 875 | Energy metabolism |
| EHF_RS02620 | ATP synthase F1, gamma subunit (*atpG*) ^5^ | 281 | Energy metabolism: ATP-proton motive force interconversion |
| EHF_RS02355 | seryl-tRNA synthetase | 423 | Protein synthesis: tRNA aminoacylation |
| EHF_RS02975 | Sensor histidine kinase/response regulator CckA ^4^ | 827 | Regulatory functions: Protein interactions |
| EHF_RS03790 | metallo-beta-lactamase family, beta-CASP subfamily | 540 | Transcription |
| EHF_RS03955 | Transcriptional repressor NrdR | 153 | Transcription: Transcription factors |
| EHF_RS01200 | heme exporter protein CcmC | 234 | Transport and binding proteins |
| EHF_RS01690 | ComEC/Rec2-related protein | 675 | Unknown function |
| EHF_RS00785 | hypothetical protein | 468 | Hypothetical proteins: Conserved |
| EHF_RS02560 | conserved domain protein | 75 | Hypothetical proteins: Conserved Domain |
| EHF_RS03455 | hypothetical protein | 501 | Hypothetical proteins: Conserved |
|  |  |  |  |

| Against *E. chaffeensis* Arkansas (22 proteins) | | | |
| --- | --- | --- | --- |
| EHF_RS00950 | Type IV Secretion Effector Etf-2, N-terminus ^[[6]](#footnote-6)^ | 137 | Cellular processes: Pathogenesis |
| EHF_RS02090 | DNA polymerase III, epsilon subunit (*dnaQ*) ^5^ | 242 | DNA metabolism: DNA replication |
| EHF_RS03660 | patatin-like phospholipase family protein ^5^ | 312 | Fatty acid and phospholipid metabolism |
| EHF_RS00305 | conserved hypothetical protein | 166 | Hypothetical proteins |
| EHF_RS00380 | conserved hypothetical protein | 319 | Hypothetical proteins |
| EHF_RS02435 | conserved hypothetical protein | 446 | Hypothetical proteins |
| EHF_RS00985 | hypothetical protein | 205 | Hypothetical proteins |
| EHF_RS01000 | hypothetical protein | 222 | Hypothetical proteins |
| EHF_RS01005 | hypothetical protein | 223 | Hypothetical proteins |
| EHF_RS01010 | hypothetical protein | 210 | Hypothetical proteins |
| EHF_RS02845 | hypothetical protein | 85 | Hypothetical proteins |
| EHF_RS03420 | hypothetical protein | 315 | Hypothetical proteins |
| EHF_RS04230 | hypothetical protein | 84 | Hypothetical proteins |

1. Proteins specific to *Ehrlichia* sp. HF were determined based on 2-way comparison analysis by Blastp algorithm (E-value < 1e^-10^) against *E. muris* subsp. *eauclairensis* Wisconsin, *E. muris* AS145, or *E. chaffeensis* Arkansas. *Ehrlichia* sp. HF-specific proteins identified in 4-way comparisons (9 proteins in Table S1) were not listed in this Table. Proteins of interest were highlighted in yellow or light gold backgrounds. [↑](#footnote-ref-1)
2. Tblastn search indicates that the homolog of *E. chaffeensis* TRP120 might be split into two pseudogenes (EMUCRT_0995 and EMUCRT_0731) present in two separate contigs (NZ_LANU01000002 and NZ_LANU01000003) of the incomplete genome sequences. [↑](#footnote-ref-2)
3. The gene encoding 3mg in *E. muris* subsp. *eauclairensis* genome contains an internal frameshift, resulting an internal stop at 99 aa and was annotated as a pseudogene. [↑](#footnote-ref-3)
4. RpoB/C were misannotated in *E. muris* subsp. *eauclairensis* genome as a concatenated pseudogene EMUCRT_RS04655. [↑](#footnote-ref-4)
5. Genes encoding GyrA, PolI, AtpG, and CckA of *E. muris* AS145 were annotated as pseudogenes due to frameshift (occurred in gene sequences with long stretches of “AA” or “TT” nucleotides). [↑](#footnote-ref-5)
6. EHF_RS00950 was matched to N-terminus of *E. chaffeensis* Arkansas Etf-2 with E-value of 1e^-07^; Homolog to EHF_RS02090 (*E. chaffeensis* *dnaQ*) contains a frameshift at AA^193^ and was labeled as annotated (but DnaQ domain is complete); Homolog to EHF_ RS03660 (*E. chaffeensis* Patatin) contains a frameshift and was annotated as a pseudogene. [↑](#footnote-ref-6)
