# Supplementary material for: Comparative Analysis of Genome of Ehrlichia sp. HF, a Model Bacterium to Study Fatal Human Ehrlichiosis
Source: BMC Genomics. 2021 Jan 6;22:11. doi: 10.1186/s12864-020-07309-z (PMC7789307; doi:10.1186/s12864-020-07309-z)
Supplement: Supplementary file 4 — Additional file 4: Table S4. Internal Repeats of Ehrlichia Tandem Repeat Proteins [file 12864_2020_7309_MOESM4_ESM.docx]

# Supplementary Table 4. Internal Repeats of *Ehrlichia* Tandem Repeat Proteins ^[[1]](#footnote-1)^

# I. TRP32 Repeats ^[[2]](#footnote-2)^

- ECH_0170 (VLPT, 198 aa, 3¼ consecutive repeats with 30 aa-length at residues 33-131):

---------------------------------------------------------------------------

**EEVIQLESDLQQSSNSDLHGSFS-VELFDPFK**

**EAV-QLGNDLQQSSDSDLHGSFS-VELFDPSK**

**EEV-QLESDLQQSSNSDLHES-SFVELPGPSK**

**EEV-QFEDD**

---------------------------------------------------------------------------

# II. TRP47 Repeats ^[[3]](#footnote-3)^

- ECH_0166 (316 aa, 7 consecutive 19-aa repeats at residues 158-290):

---------------------------------------------------------------------------

**ASVSEGDAVVNAVSQEAPA**

**ASVSEGDAVVNAVSQETPA**

**ASVSEGDAVVNAVSQETPA**

**ASVSEGDAVVNAVSQETPA**

**ASVSEGDAVVNAVSQETPA**

**ASVSEGDAVVNAVSQETPA**

**ASVSEGDAVVNAVSQETPA**

---------------------------------------------------------------------------

# III. TRP120 Repeats ^[[4]](#footnote-4)^

- ECH_0039 (548 aa, 4⅓ consecutive repeats with 80 aa-length at residues 79-424):

---------------------------------------------------------------------------

**VSQPSLEPFVAESEVSKVEQEKTNPEVLIKDLQDVASHESGVSDQPAQVVTERENEIESHQGETEKESGITESHQKEDEI**

**VSQSSSEPFVAESEVSKVEQEETNPEVLIKDLQDVASHESGVSDQPAQVVTERESEIESHQGETEKESGITESHQKEDEI**

**VSQPSSEPFVAESEVSKVEQEETNPEVLIKDLQDVASHESGVSDQPAQVVTERESEIESHQGETEKESGITESHQKEDEI**

**VSQPSSEPFVAESEVSKVEQEETNPEVLIKDLQDVASHESGVSDQPAQVVTERESEIESHQGETEKESGITESHQKEDEI**

**VSQPSSEPFVAESEVSKVEQEKTNPE**

---------------------------------------------------------------------------

- EHF_RS04475/EHF_0993 (584 aa, 4¼ consecutive repeats with 100 aa-length at residues 30-455):

---------------------------------------------------------------------------

**AILGDSKNNPVVEAGDHSGTHQEGNEIISQPSSAPAASVIAEEEISKTHQGEDNLKEDLHSAVTSDNLVTLDQTVPVSVGGEKEISETHQGQNLLNMIMH**

**AILGDSKNNPVVEAGGHSGTHQEGNEIIFQPSSAPAASVIAEEEISKTHQGEDNLKEDLHSAVTSDNLVILDQTVPVSVGGEKEISETHQGQNLLNMIMH**

**AILDDSNNNPVVEAGDHSGTHQEGNEIIFQPSSAPAASVIAEEEISKTHQGEDNLKEDLHSAVTSDNLVILDQTVPVSVGGEKEISETHQGQNLLNMIMH**

**AILDDSNNNPVVEAGDHSGTHQEGNETIFQPSSAPAASVIAEEEISKTHQGEDNLKEDLHSAVTSDNLVTLDQTVPVSLGGEKEISETHQGQNLLNMIMH**

**AILDDSNNNPVVEAGDHSGTHQEGNE**

---------------------------------------------------------------------------

- **EMUR**_**00035** (1,288 aa, 12.4 repeats with 56~67-aa length at residues 169-1176):

---------------------------------------------------------------------------

Rpt1: **HQEENETISQPSSEPAASVVIGEEKEISETHFESDNPKVSKEENLHADVVSDNLDQAVPVVAEVSSA**

Rpt1: **HQEENETISQPSLEPAISVVIEEEKEISETHLESDNPKVSKEENLHADVVSDNLDQAVPVVAEVSSA**

Rpt1: **HQEENETISQPSSEPAISVVTEEEKEISETHLESDNPKVSKEEDLHADVVSDNLDQAISVVTEEEVS**

Rpt1: **HREENETISQPSSEPAISVVTEEEKEISETHLESDNPKVSKEEDLHADVVSDNLDQAISVVTEEEVS**

Rpt1: **--------SQPSLESAASVVTEEEKEISETHLESDNPKVSKEEDLHADVVSDNLDQADSVVTEETSA**

Rpt2: **HREENETISQPSSEPAISVVTEEEVSGQNLLDILVRTIWGSSKDSNVVEVEDHFGT**

Rpt1: **HQEENETISQPSSEPAASVVIGEEKEISETHFESDNPKVSKEENLHADVVSDNLDQAVPVVAEVSSA**

Rpt1: **HQEENETISQPSLEPAISVVIEEEKEISETHLESDNPKVSKEENLHADVVSDNLDQAVPVVAEVSSA**

Rpt1: **HQEENETISQPSLEPAISVVIEEEKEISETHLESDNPKVSKEEDLHADVVSDNLDQADSVVTEETSA**

Rpt2: **HREENETISQPSSEPAISVVTEEEVSGQNLLDILVRAISGSSKDSNVVAVEDHFGT**
Rpt2: **HQEGDKTISQPSSEPAASVVTEEEVSGQNLLDILVRAISGSSKDSNVVEVEDHFGT**

Rpt2: **HQEENETISQPSLESAISVVTEEEVSGQNLLDILVRAISGSSKDSNVVEVEDHFGT**

Rpt2: **HQEENETISQPSLESAISVVIEEE**

---------------------------------------------------------------------------

- *E. muris* subsp. *eauclairensis* Wisconsin

EMUCRT_0995 [440 aa, 5 repeats (rpt1) with 65-aa length and 2 repeats (rpt2) with 38-aa length at residues 41-440]:

---------------------------------------------------------------------------

Rpt1: **DQAASVVTEETSKTHQEGDKTVSQSSSELVVTEEGKEISETHLENDNPEVLIEEDLHAAVVSDNL**

Rpt1: **DQAAPVVTEETSKTHQEG-ETVSQSSSELVVTEEGKEISETHLENDNPEVLIEEDLHAAVVSDNL**

Rpt2: DQAVPAVTEEEVSEQNLLDMLVRAILGSSKDSDVVEVE

Rpt1: **DQAASVVTEETSKTHQEGDKTVSQSSSELVVTEEGKEISETHLENDNPEVLIEEDLHAAVVSDNL**

Rpt2: DQAVPAVTEEEVSEQNLLDMLVRAILGSSKDSDVVEVE

Rpt1: **DQAASVVTEETSKTHQEGDKTVSQSSSELVVAEEGKEISETHLENDNPEVLIEEDLHAAVVSDNL**

Rpt1: **DQAASVVTEETSKTHQEGDKTVSQSSSELVVTEEGKEISETHLENDNPEVLIEEDLHAAVVSDNL**

---------------------------------------------------------------------------

EMUCRT_0731 [341 aa, 3.9 repeats (rpt1) with 65-aa length at residues 1-252]:

---------------------------------------------------------------------------

Rpt1: **DQAASVVTEETSKTHQEGDKTVSQSSSELVVTEEGKEISETHLENDNPEVLIEEDLHAAVVSDNL**

Rpt1: **DQAASVVTEETSKTHQEGDKTVSQSSSELVVTEEGKEISETHLENDNPEVLIEEDLHAAVVSDNL**

Rpt1: **DQAASVVTEETSKTHQEG-ETVSQSSSELVVTEEGKEISETHLENDNPEVLIEEDLHAAVVSDNL**

Rpt1: **DQAVPVVTEETSKTHQEG-ETVSQSSSELVVTEEEKEISETHLENDNPKFLIEEDLH**

---------------------------------------------------------------------------

1. TRP Homologs of *E. chaffeensis* Arkansas were identified using Blastp among *Ehrlichia* spp., and the internal tandem repeats were determined by XSTREAM (https://amnewmanlab.stanford.edu/xstream/) (Newman, 2007). [↑](#footnote-ref-1)
2. *E. chaffeensis* TRP32 (ECH_0170) protein (or variable length PCR target/VLPT) contains 3¼ consecutive 30-aa VLPT repeats. However, no repeats or VLPT domains were detected in other *Ehrlichia* spp*.* [↑](#footnote-ref-2)
3. *E. chaffeensis* TRP47 protein (ECH_0166) contains seven consecutive 19-aa repeats at its C-terminus. However, no repeats were detected in TRP47 homologs of other *Ehrlichia* spp. *(E. muris* subsp. *eauclairensis*, EMUCRT_0637; *E. muris* subsp. *muris*, EMUR_0500; *Ehrlichia sp.* HF, EHF_0897, 255 aa). [↑](#footnote-ref-3)
4. *E. chaffeensis* TRP120 protein (ECH_0039) contains 4⅓ repeats with 80 aa-length. TRP120 homolog in *Ehrlichia sp.* HF (EHF_0897/EHF_RS04625, 584 aa) contains 4¼ repeats with 100 aa-length; a much larger protein was identified in *E. muris* AS145 (EMUR_0035/MR76_RS00035, 1,288 aa), which has 12.4 repeats with different repeat lengths (rpt1, 67-aa; rpt2, 56-aa). The homolog of *E. chaffeensis* TRP120 in *E. muris* subsp. *eauclairensis* Wisconsin might be split into two pseudogenes (EMUCRT_0995 and EMUCRT_0731) present in two separate contigs (NZ_LANU01000002 and NZ_LANU01000003) of the incomplete genome sequences, which has a total of ~11 repeats with different repeat lengths (rpt1, 65-aa; rpt2, 38-aa). Colored backgrounds indicate different repeat sequences. [↑](#footnote-ref-4)
