# Supplementary material for: Comparative Analysis of Genome of Ehrlichia sp. HF, a Model Bacterium to Study Fatal Human Ehrlichiosis
Source: BMC Genomics. 2021 Jan 6;22:11. doi: 10.1186/s12864-020-07309-z (PMC7789307; doi:10.1186/s12864-020-07309-z)
Supplement: Supplementary file 5 — Additional file 5: Table S5. Primers used in this study [file 12864_2020_7309_MOESM5_ESM.docx]

# Supplementary Table 5. Primers used in this study

| **Primer Target** | **Primer name and sequence** | **Product size** | **Literature** |
| --- | --- | --- | --- |
| *Ehrlichia* sp. HF 16S rRNA gene | HF51f: AAGTCGAACGGACAATTACC  HF954r: GTTAGGGGGATACGACCTTC | 923 bp | [126] |
| Canine G3PDH DNA | F: GAACGGGAAGCTCACTGGCATGGC  R: TGAGGTCCACCACCCGGTTGCTG | 310 bp | [129] |
