# Supplementary figures and images for: Comparative Analysis of Genome of Ehrlichia sp. HF, a Model Bacterium to Study Fatal Human Ehrlichiosis
Source: BMC Genomics. 2021 Jan 6;22:11. doi: 10.1186/s12864-020-07309-z (PMC7789307; doi:10.1186/s12864-020-07309-z)

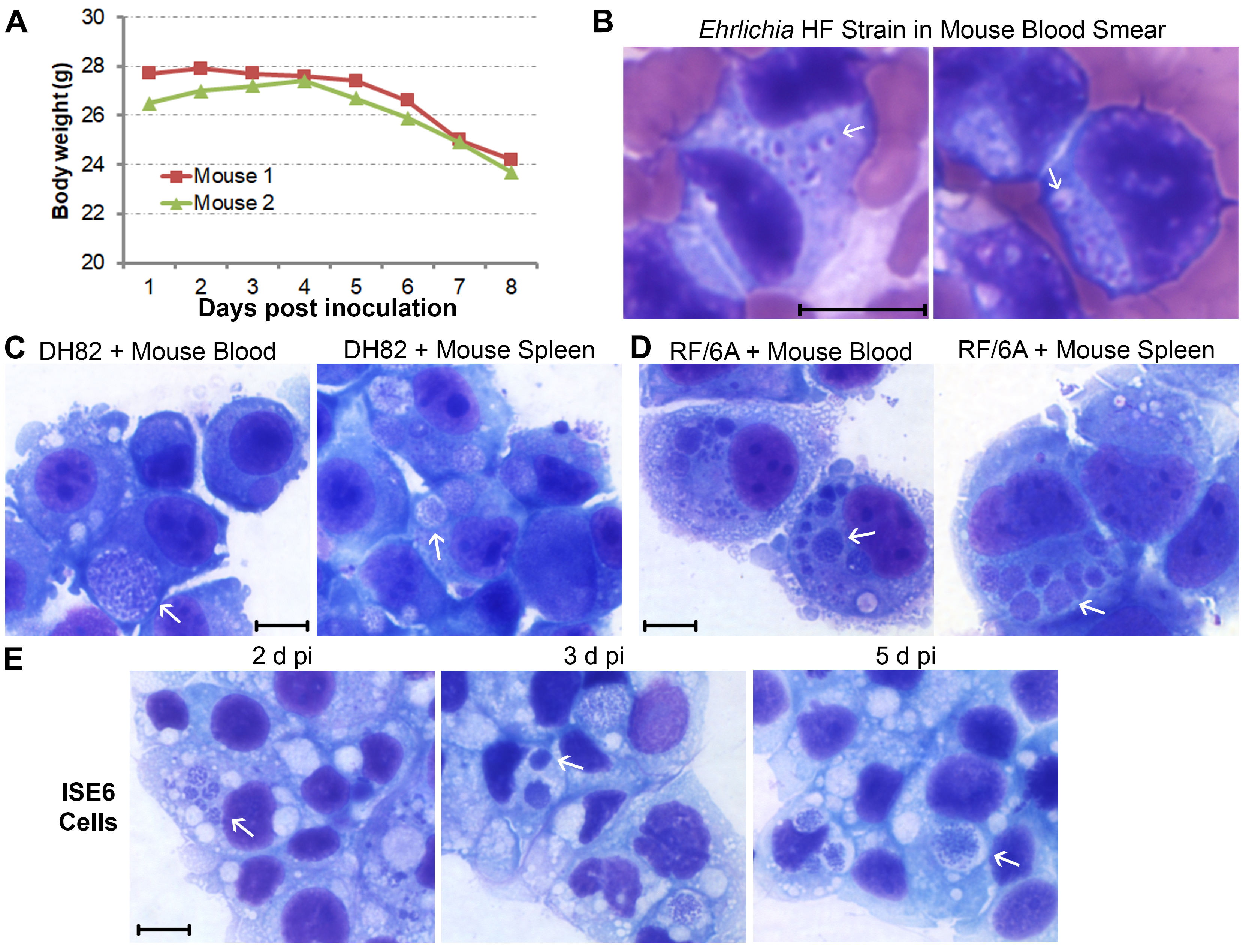

Supplement: Supplementary file 6 — Additional file 6: Figure S1. Culture Isolation of Ehrlichia sp. HF from infected mouse buffy coat and spleen. (A) Body weight of mice inoculated with mouse spleen homogenates containing Ehrlichia sp. HF following days post inoculation. (B) Ehrlichia sp. HF (white arrows) in the blood monocytes from buffy coat smear by Diff-Quik staining. (C-D) Large Ehrlichia-containing inclusions (white arrows) in DH82 cells at ~ 3 weeks post infection (pi) or RF/6A cells at 2 weeks pi. (E) ISE6 cells were infected with purified host cell-free Ehrlichia sp. HF-infected DH82 cells and cultured in L15C300 media at 34°C. Infectivity reached 10% at 3 - 5 d pi with large morulae packed with Ehrlichia. Bar, 10 μm. [file 12864_2020_7309_MOESM6_ESM.tif]

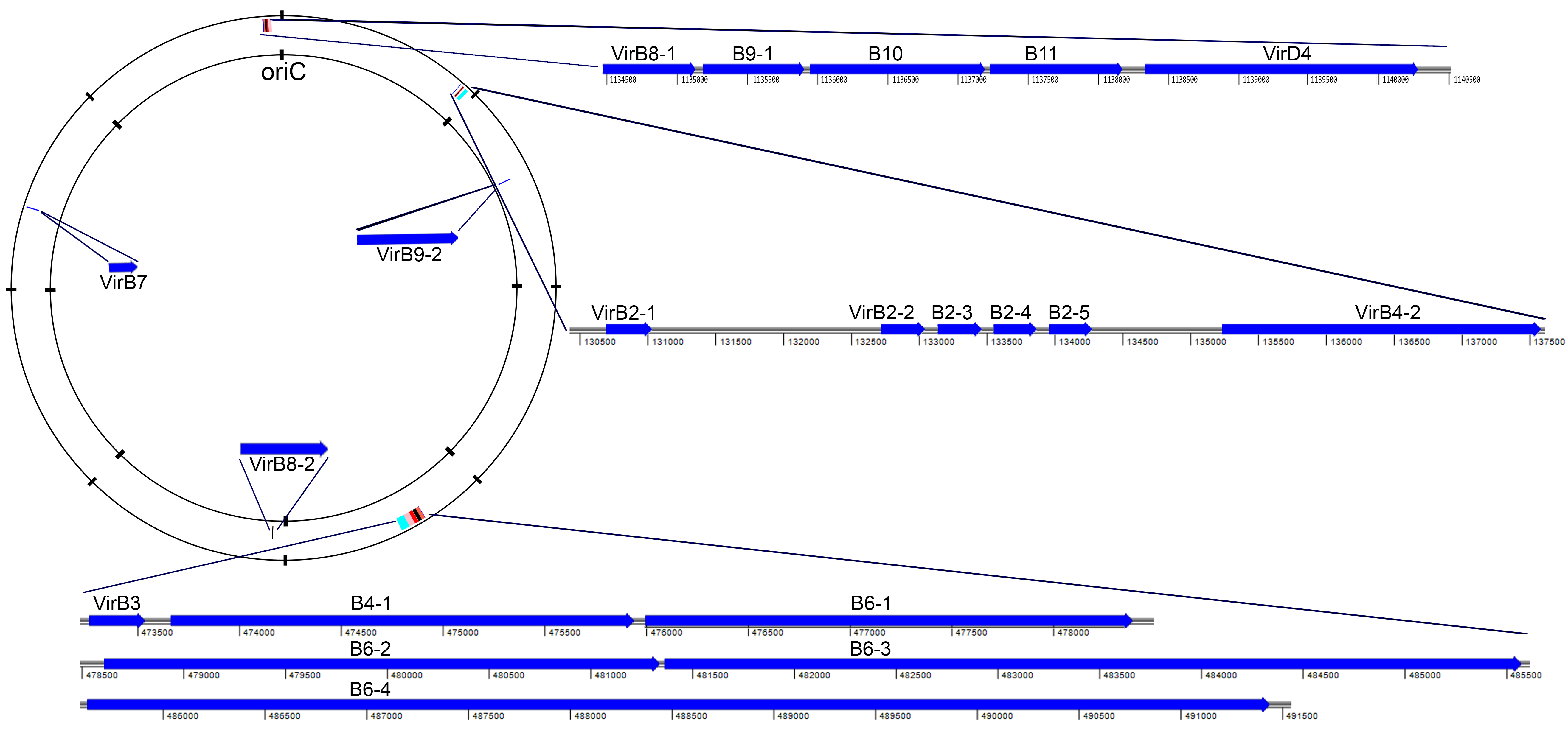

Supplement: Supplementary file 7 — Additional file 7: Figure S2. Gene Structures of Ehrlichia sp. HF Type IV Secretion System. Ehrlichia sp. HF encodes a Type IV secretion system. These virB/D genes are split into three major operons: virB2/4, virB3/4/6virB3/4/6, virB8/9/10/11/D4, and three separate loci: virB7 and duplicated virB8-2 and virB9-2. virB2 genes are duplicated into 5 copies, whereas virB6 into 4 copies. Genes encoding virB1 and virB5 are not present in HF genome. Note: Due to the short protein length and low homology, virB7 was not annotated as an ORF by NCBI automated annotation pipeline. However, by TBLASTN using A. marginale VirB7 protein sequence [121] against the entire HF genome sequence, a putative virB7 gene was identified and manual curated. [file 12864_2020_7309_MOESM7_ESM.tif]

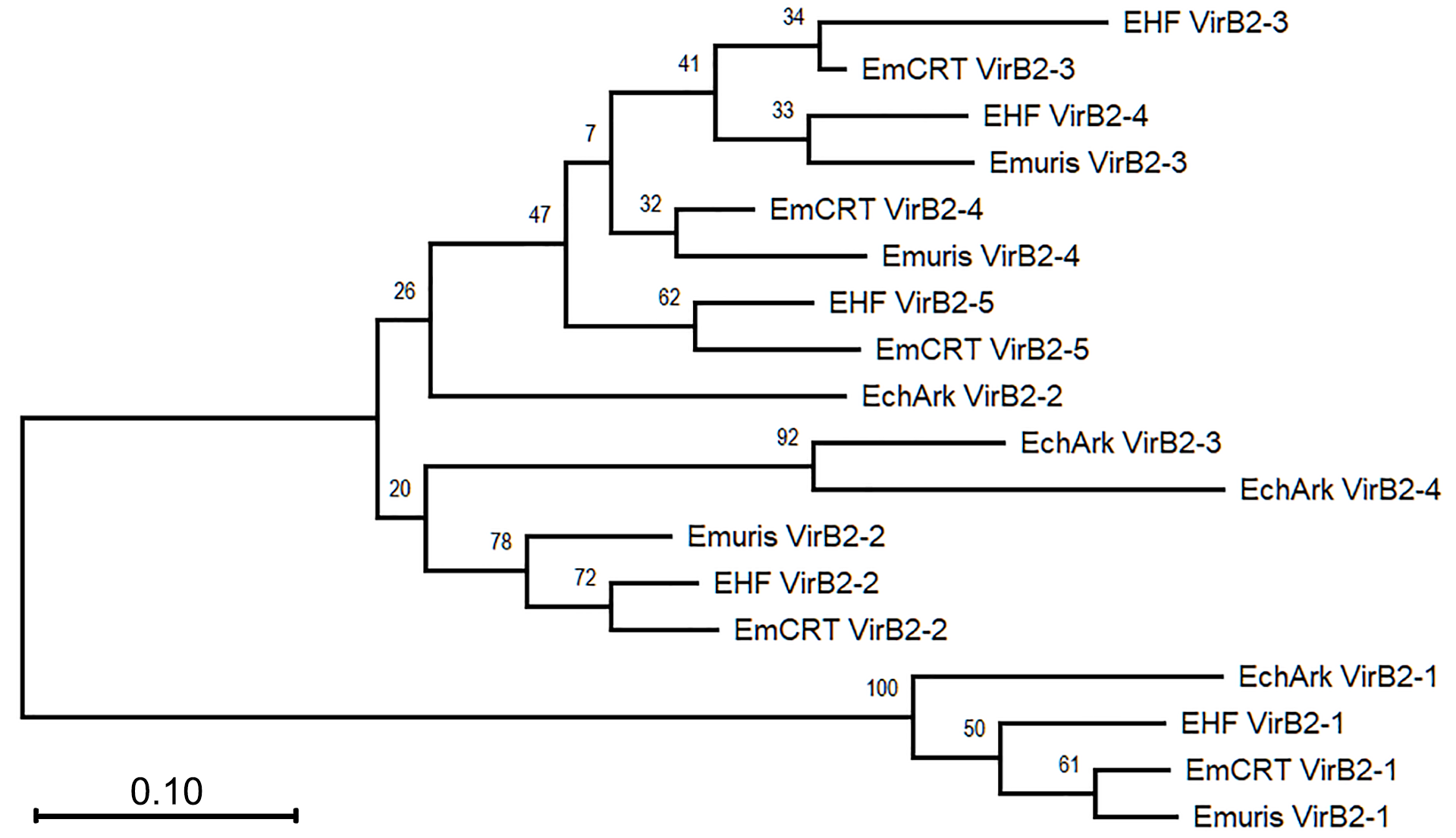

Supplement: Supplementary file 8 — Additional file 8: Figure S3. Phylogenetic analysis of Ehrlichia VirB2 paralogs of representative Ehrlichia species. Phylogenic tree of VirB2 paralogs of representative Ehrlichia species, including Ehrlichia sp. HF (EHF), E. chaffeensis Arkansas (EchArk), E. muris subsp. muris AS145 (Emuris), and E. muris subsp. eauclairensis Wisconsin (EmCRT). The evolutionary history was inferred by using the Maximum Likelihood method and JTT matrix-based model, and the tree with the highest log likelihood is shown. The tree is drawn to scale with branch lengths measured in the number of substitutions per site (below branches), and the percentage of trees in which the associated taxa clustered together is shown above the branches. Evolutionary analyses were conducted in MEGA X. [file 12864_2020_7309_MOESM8_ESM.tif]

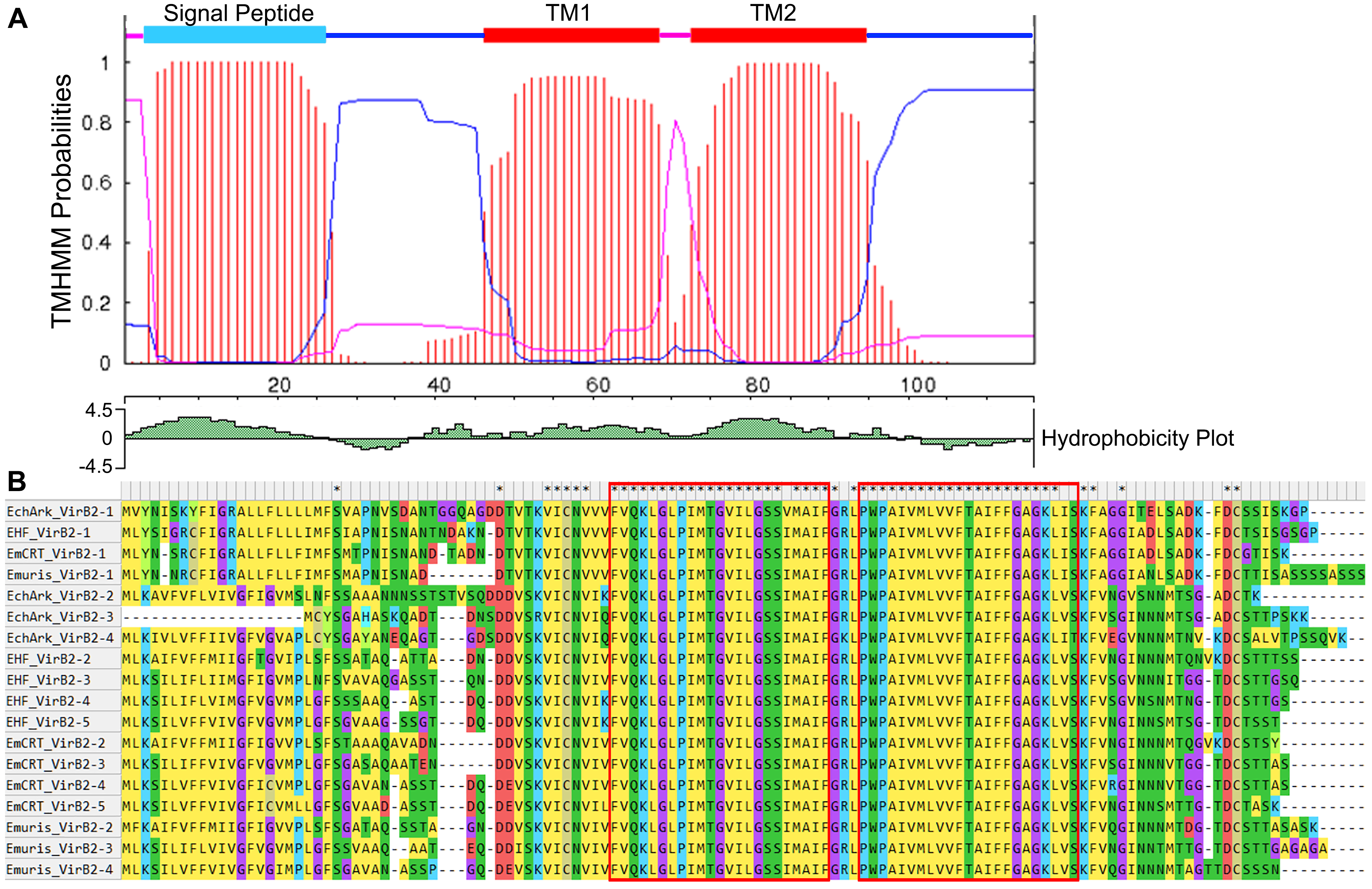

Supplement: Supplementary file 9 — Additional file 9: Figure S4. Domain structures and alignment of VirB2 paralogs of representative Ehrlichia species (A) Domain structures of Ehrlichia sp. HF VirB2-4. Analysis of Ehrlichia sp. HF VirB2-4 showed that it possesses a signal peptide (cleavage site between residues 29 and 30) and two putative transmembrane motifs. The signal peptide and transmembrane helices (TM) were predicted by SignalP-5.0 Server (http://www.cbs.dtu.dk/services/SignalP/) and TMHMM Server 2.0 (http://www.cbs.dtu.dk/services/TMHMM/), respectively. Hydrophobicity was analyzed by Protean program (DNAStar). (B) Alignment of VirB2 paralogs of representative Ehrlichia species showed that although these proteins are more divergent on the N- and C-terminus, they are highly conserved in the central transmembrane motifs or hydrophobic regions (indicated by red boxes). *, conserved among all Ehrlichia VirB2 proteins. [file 12864_2020_7309_MOESM9_ESM.tif]

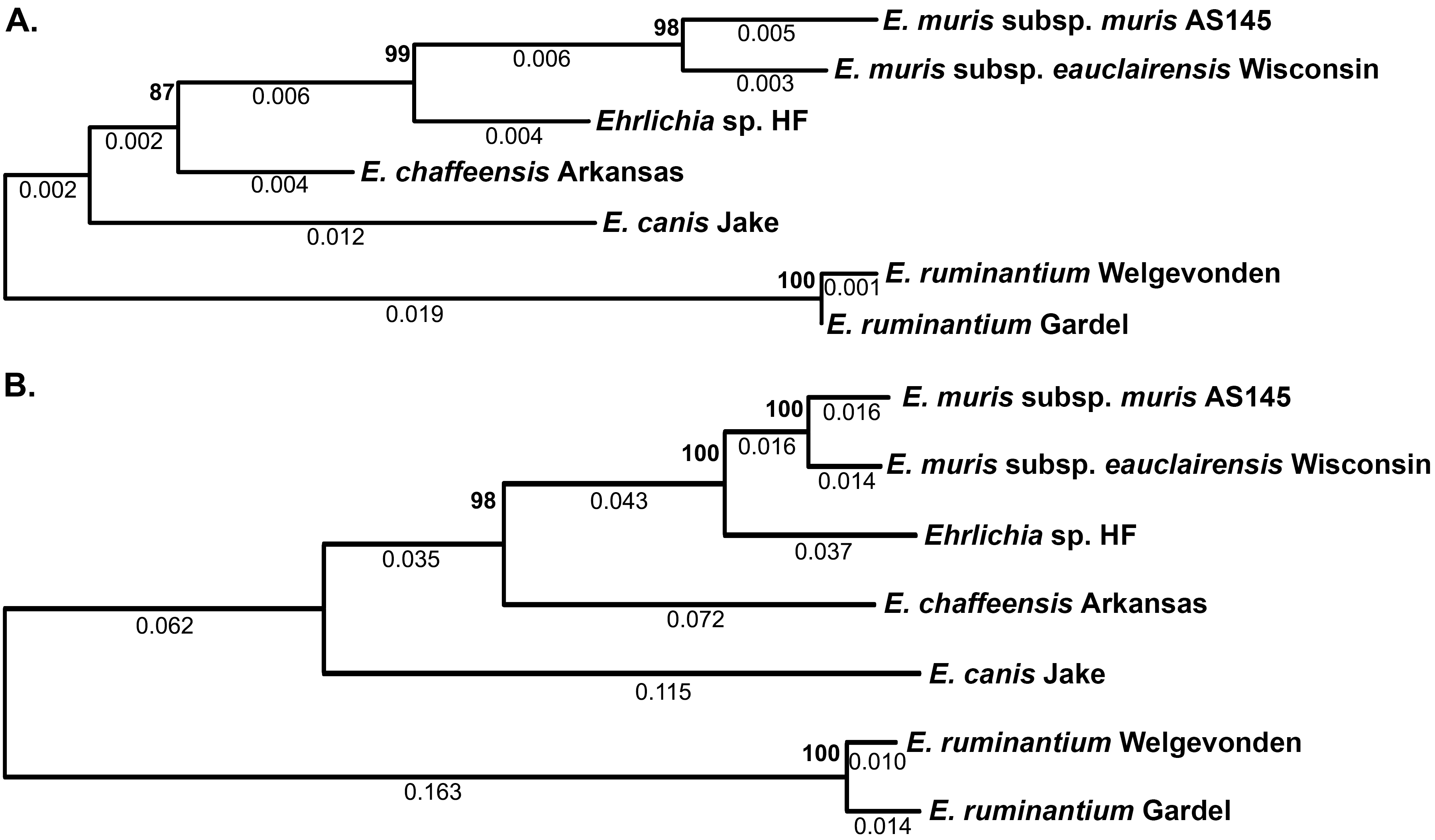

Supplement: Supplementary file 10 — Additional file 10: Figure S5. Phylogenetic trees of representative Ehrlichia species based on 16S rRNA sequences and concatenated protein sequences. (A) 16S rRNA genes from seven representative Ehrlichia spp. were aligned individually using MegAlign (1,514 nucleotides of aligned nucleotides). (B) Eight Ehrlichia proteins, including 5 conserved housekeeping proteins (TyrB, Mdh, Adk, FumC, and GroEL) and 3 divergent outer membranes proteins (P28, VirB2-1, and VirB6-1) from these Ehrlichia spp. were aligned using MegAlign. The aligned protein sequences were trimmed and concatenated (3,188 AA total). The evolutionary analyses were inferred by using the Maximum Likelihood method and Tamura-Nei model for 16S rRNA, or JTT matrix-based model for concatenated proteins. Bootstrap values for 1,000 replicates were obtained using MEGA X. The trees with the highest log likelihood (-2609.17 for 16S rRNA, and -17382.50 for proteins) were shown, and the percentage of trees in which the associated taxa clustered together in the bootstrap test was shown above each branch. The tree is drawn to scale with branch lengths measured in the number of average nucleotide substitutions per site (shown under each branch). GenBank Accession numbers for seven representative Ehrlichia spp.: Ehrlichia sp. HF, NZ_CP007474.1; E. chaffeensis Arkansas, NC_007799.1; E. muris subsp. muris AS145, NC_023063.1; E. muris subsp. eauclairensis Wisconsin, LANU01000000; E. canis Jake, NC_007354.1; E. ruminantium Welgevonden, NC_005295.2; E. ruminantium Gardel, NC_006831.1. [file 12864_2020_7309_MOESM10_ESM.tif]

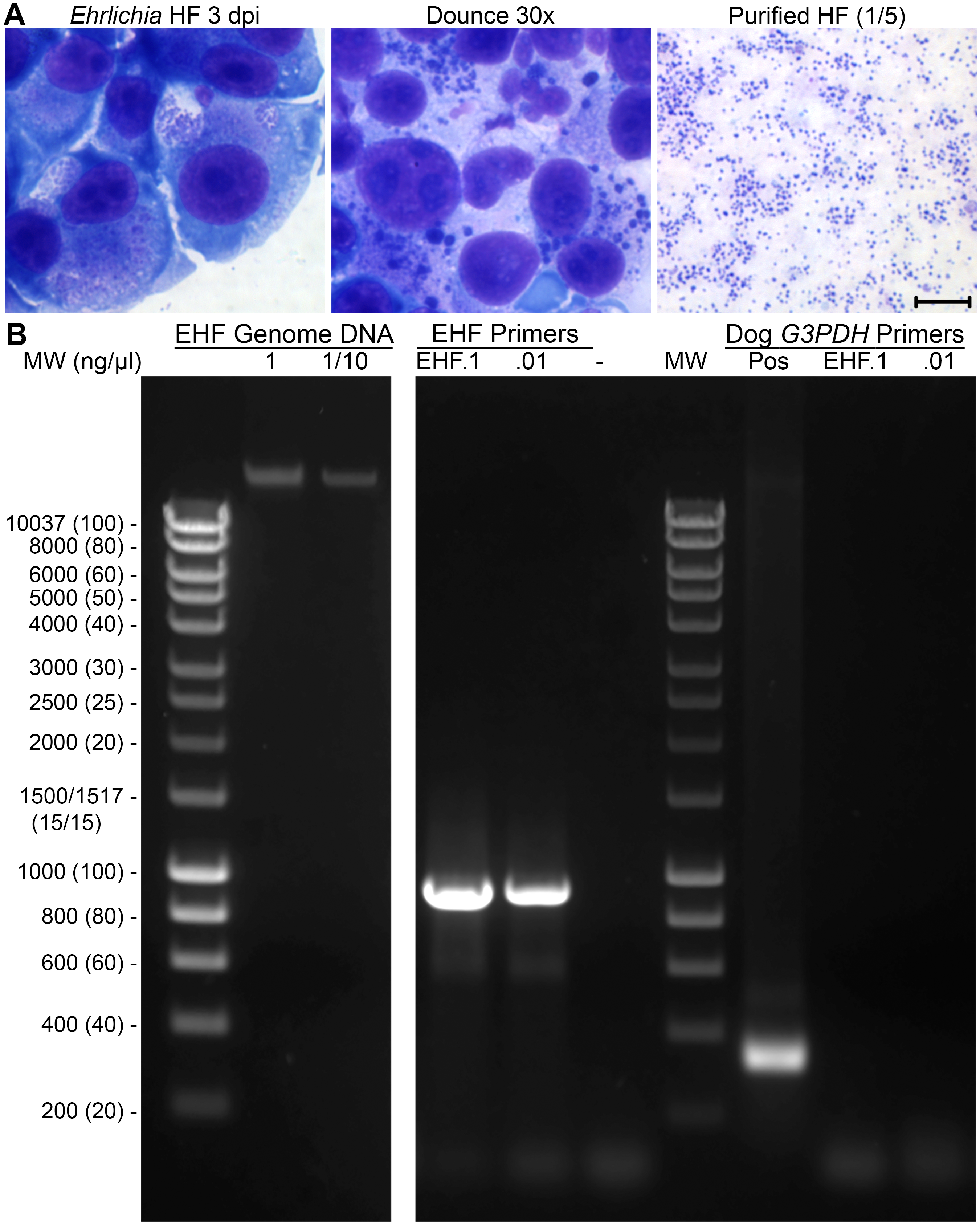

Supplement: Supplementary file 11 — Additional file 11: Figure S6. Purification of host cell-free Ehrlichia sp. HF and bacterial genomic DNA. (A) Twelve T175 flasks of Ehrlichia sp. HF-infected DH82 cells (>80% infectivity) at 3d pi were homogenized in 30 ml of 1× SPK for 30 times with type B tight-fitting pestle. Pellet following centrifugation at 700 × g was homogenized for additional 30 times. Both homogenates were step-wise centrifuged at 700, 1,000, and 1,500 × g, passed through 5.0- and 2.7-μm filters, and centrifuged at 10,000 × g for 10 min. Host cell-free Ehrlichia sp. HF was purified with very low host nuclear contamination under Diff-Quik staining. Bar, 10 μm. (B) Genome DNAs of Ehrlichia sp. HF (EHF) were purified using Qiagen genomic tips and dissolved in TE buffer. DNAs were resolved using 0.9% agarose with BioLine molecular weight (MW) markers with DNA concentrations of each band showing inside parenthesis. Genomic DNA bands above 20 kB were visible, and the concentration was above 15 ng/μl. PCR reactions were carried out with 35 cycles at 98°C for 30-second, 60°C for 30-second, and 68°C for 1-minute. Primers targeting 16S rRNA gene of Ehrlichia sp. HF detected specific bands at 1/100 dilutions, but dog G3PDH primers did not amplify any bands under any dilutions (Dilutions: .1, 1/10, .01, 1/100 dilution; Pos.: positive control using DNA isolated from dog DH82 cells; -, negative control without DNA input). [file 12864_2020_7309_MOESM11_ESM.tif]
